# The role of redox signaling in mitochondria and endoplasmic reticulum regulation in kidney diseases

**DOI:** 10.1007/s00204-025-04041-z

**Published:** 2025-04-11

**Authors:** Omar Emiliano Aparicio-Trejo, Estefani Yaquelin Hernández-Cruz, Laura María Reyes-Fermín, Zeltzin Alejandra Ceja-Galicia, José Pedraza-Chaverri

**Affiliations:** 1 Department of Cardio-Renal Physiopathology, National Institute of Cardiology Ignacio Chávez, 14080 Mexico City, Mexico; 2 Laboratory F-315, Department of Biology, Faculty of Chemistry, National Autonomous University of Mexico, Mexico City, Mexico; 3 Department of Cardiovascular Biomedicine, National Institute of Cardiology Ignacio Chávez, 14080 Mexico City, Mexico

**Keywords:** ROS signaling, Mitochondria redox regulation, Mitochondria in renal disease, Mitochondrial biogenesis and b-oxidation, Endoplasmic reticulum stress redox regulation

## Abstract

Kidney diseases are among the fastest worldwide growing pathologies. This growth together with their high mortality rate emphasizes the importance of generating vital information about the mechanism involved in their pathophysiology to determine possible therapeutic targets. Recently, mitochondrial damage and their implication in the reactive oxygen spices (ROS) signaling and redox homeostasis have emerged as a hub point in the pathologic mechanism involved in renal pathologies. ROS in low levels are necessary to maintain cell processes as well as the mitochondria homeostasis and its association with other organelles, especially the with the endoplasmic reticulum (ER). However, the information about how redox signaling interacts and interferes with other cellular processes and the mechanism involved has not been fully integrated. Furthermore, in higher concentrations, these ROS promotes pathologic pathways linked to renal disease progression like, mitochondrial biogenesis reduction, ER stress, calcium overload, inflammation, cell death and fibrosis. Therefore, the aim of this review is to describe the molecular mechanisms involved in the redox signaling influence on mitochondrial and ER homeostasis, focusing on lipid metabolism and ß-oxidation, mitochondrial biogenesis, inflammations, ER stress and calcium homeostasis, as well as the effects of these alteration in the genesis and development of renal disease, with emphasis in acute kidney injury (AKI) and chronic kidney disease (CKD).

## Introduction

Kidney disease, together with diabetes, has been the fastest worldwide growing pathology among non-communicable diseases in recent years; the World Health Organization (WHO) recently considered them a global pandemic of prioritized importance, which also increases the risk of development of other pathologies like cardiovascular disease, infarct, neurovascular disease, diabetes, between others (Francis et al. 2024; Nephrology 2024). Chronic kidney disease (CKD) englobes diverse pathologies characterized by the gradual deterioration of kidney function over time (Carrero et al. 2017; Francis et al. 2024). On the other hand, Acute Kidney Injury (AKI) englobes several pathologies characterized by the quick deterioration of renal functions in a relatively short time interval (Kellum et al. 2021). Several evidence showed that intense episodes of AKI or a series of them may result in CKD development. Likewise, CKD is widely recognized as a risk factor for AKI episodes (Hsu and Hsu 2016; Kellum et al. 2021). The high prevalence of these two diseases, together with their close interconnection, made them have high prevalence, morbidity, mortality, and costs to the health system in the world. Furthermore, although several current therapeutic approximations allow CKD progression, alternative approaches are still necessary to reduce morbidity and mortality (Feng et al. 2017; Nephrology 2024). This highlights the necessity of understanding the complex mechanisms involved in generating and progressing these renal diseases.

On the other hand, mitochondrial dysfunction and redox state have emerged as a hub in the pathologic mechanism involved in renal pathologies (Bhargava and Schnellmann 2017; Forbes and Thorburn 2018; Lumpuy-Castillo et al. 2024). Kidney function highly depends of the homeostasis of this organelle, and mitochondria play a hub in signaling pathways, metabolic regulation, calcium homeostasis, and inflammation through the redox regulation, which also is important for the feedback communication with other organelles like endoplasmic reticulum (ER) and peroxisomes (Martínez‐Klimova et al. 2020; Lumpuy-Castillo et al. 2024). In addition, the reactive oxygen species (ROS) produced by these organelles may act in certain physiologic concentrations as second messengers necessary to maintain cellular homeostasis, making these ROS molecules able to regulate signaling pathways like fibrosis, cell death, and inflammation (Holmström and Finkel 2014). Therefore, mitochondrial and ER dysfunction, together with redox signaling impairment, are central to the development and progression of AKI to CKD. This review delves into the redox signaling pathways associated with mitochondrial and ER-derived ROS and their role in AKI development and CKD progression.

## ROS and the distinction between good stress and bad stress

**Fig. 1:**
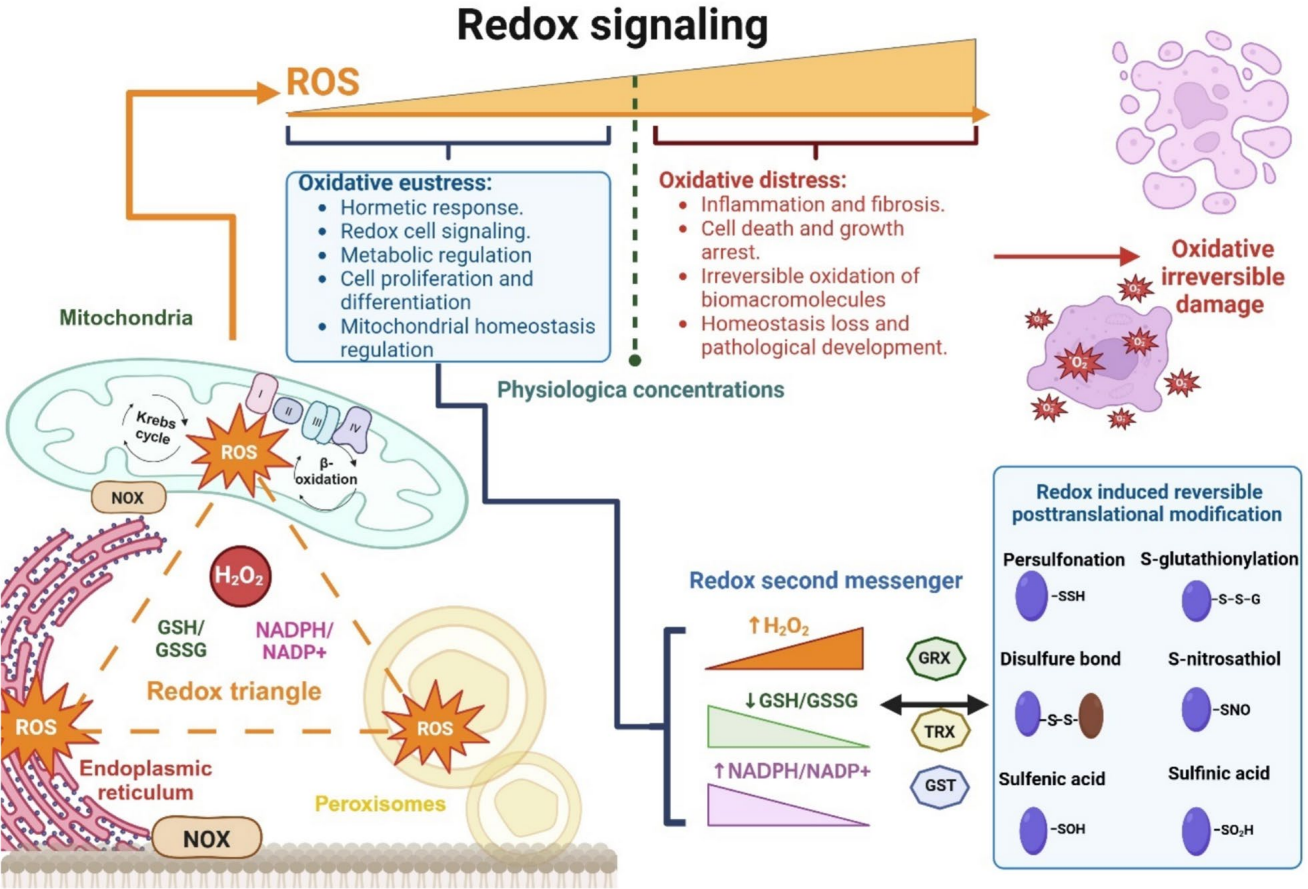
Redox signaling and triangle. The mitochondrial, endoplasmic reticulum, and peroxisomes conform to one of the main hubs in redox regulation inside the cell, known as the *redox triangle*. Under physiologic conditions, the three organelles normally produce ROS maintained in specific concentrations by the corresponding antioxidant systems. Certain ROS, like hydrogen peroxide (H_2_O_2_) acts as a second messenger that is coregulated with the reductive power of the NADPH/NADP^+^ and GSH/GSSG ratios. Small increments in H_2_O_2_ levels induce the induce an adaptive response known as “*oxidative eustress*”, in which the changes in NADPH/NADP^+^ and GSH/GSSG ratios trigger those enzymes of the TRX, GPX, and GST families catalyze redox-sensitive post-translational modification in Cys residues of the specific proteins, modifying their activity, localization and/or stability to induce an hormetic response. In contrast, when higher ROS concentration exceeds physiological levels, ROS concentration triggers aberrant and unspecific oxidations of several biomacromolecules, inducing ‘*oxidative distress*”. This process disrupts cell homeostasis and is classically associated with oxidative stress, which is present in several pathologic processes like chronic inflammation and fibrosis and is strongly linked to renal pathologies. *I* Complex I, *II* Complex II, *III* Complex III, *IV* Complex IV, *GSH* reduced glutathione, *GSSG* oxidized glutathione, *GRX* glutaredoxin, *GST* glutathione S transferase, *NADP*^*+*^ nicotinamide adenine dinucleotide phosphate oxidized form, *NADPH* nicotinamide adenine dinucleotide phosphate reduced form, *NOX* NADPH oxidase, *TRX* thioredoxin. Figure created using BioRender.com

Historically, oxidative stress has been associated with a biologic state characterized by an imbalance between ROS production and their detoxification by enzymatic and non-enzymatic systems, disrupting cellular signaling and redox control (Sies et al. 2017). However, under normal conditions, ROS at low concentrations act as second messengers and play a physiological role in intracellular signaling (Aparicio-Trejo et al. 2020b). This is especially important in mitochondria, the ER, and the peroxisomes (Fig. 1), forming what is known as the organelle redox triangle, that coordinates cell redox pathways (Jiménez-Uribe et al. 2021). ROS are reactive oxygen-containing molecules classified into free radicals and non-radical species. Among free radicals are the superoxide anion (O₂•-), hydroxyl radical (•OH), peroxyl radical (ROO•), and alkoxyl radical (RO•), with •OH being one of the most reactive and less associated with cell signaling pathways species. Non-radical species include hydrogen peroxide (H₂O₂), singlet oxygen (^1^O₂), an excited form of oxygen that triggers oxidative reactions, peroxynitrite (ONOO−), which forms from the reaction of nitric oxide (NO) with O₂•-, and hypochlorous acid (HOCl), produced by myeloperoxidase in the presence of chlorine (Domej et al. 2014). At high concentrations, all these species, both radical and non-radical, may significantly affect cellular macromolecules by unspecific oxidation. For instance, ONOO– induces nitration of tyrosine residues in proteins, altering their function, while HOCl can oxidize lipids and membrane proteins (Zavodnik et al. 2001; Ferrer-Sueta et al. 2018; Hawkins 2020).

The term oxidative stress is widely used to describe the imbalance of oxidants over antioxidants, in which their accumulation causes damage to macromolecules such as proteins, lipids, and nucleic acids, contributing to pathologic processes such as aging and chronic diseases (Bhat et al. 2015; Kowalczyk et al. 2021). However, as is shown in Fig. 1 nowadays, it necessarily makes a distinction between good stress, in which the increase in specific ROS concentrations induces an adaptive response known as “oxidative eustress”, and bad stress, known as “oxidative distress”, in which higher ROS concentration triggers aberrant and unspecific target oxidations, disrupting cell homeostasis (Aparicio-Trejo et al. 2020a, b). For example, (H_2_O_2_ at physiologic concentrations is currently considered a redox signaling agent more than a dangerous side product (Lumpuy-Castillo et al. 2024). In addition, the value of H_2_O_2_ physiologic concentration changes between the differences cell compartments and organelles, being in the cytosol from approximately 80 nM, in the mitochondrial matrix from 5 to 20 nM and 700 nM in the ER lumen (Wei et al. 2014; Liu et al. 2017; Forbes and Thorburn 2018). Therefore, each compartment has its “redox tone”, corresponding to the normal ROS concentrations that maintain cell homeostasis (Tanada et al. 2017; Hallan et al. 2017; Harzandi et al. 2021). The H_2_O_2_ scavenger and signaling transduction are mediated and amplified by the oxidation of the glutathione (GSH) (Aparicio-Trejo et al. 2020a; Lumpuy-Castillo et al. 2024), thus the GSH equilibrium with their oxidized form (GSSG), also known as GSH/GSSG ratio is also a key regulator of the redox tone and can be measured by the determination of glutathione redox potential (*E*_GSH/GSSG_) (Aparicio-Trejo et al. 2020a; Jung et al. 2024). Furthermore, GSH is the most abundant low-molecular-weight antioxidant in the cell with intracellular concentration from 10 to 30 mM (Liu et al. 2018; Kamarauskaite et al. 2020), and it’s also highly compartmentalized, with independent cytosol, ER, mitochondrial and peroxisome concentrations and GSH/GSSG ratio (Stallons et al. 2014; Kang et al. 2015; Hallan et al. 2017; Jung et al. 2024), making them and a good candidate in redox cell signaling amplification (Aparicio-Trejo et al. 2020a). On the other hand, when H_2_O_2_ levels reach 100 nM or higher in the cytosol, or the GSH/GSSG ratio is low, these promote oxidative distress and cell damage (Tanada et al. 2017; Hallan et al. 2017). Although it has been less widespread in the literature, low levels or the lack of ROS also disrupt the physiologic redox signaling, leading to cell damage and metabolism disruption in a process known as “reductive distress” (Stadler et al. 2015).

The mitochondrial electron transport chain (ETC) within the cell is the primary source of ROS production during aerobic respiration. Approximately 1–2% of the absorbed oxygen is converted to ROS, especially in the redox center of mitochondrial complexes, dehydrogenase, and cofactors involved in the ETC and Krebs cycle (Sena and Chandel 2012; Zorov et al. 2014; Kowalczyk et al. 2021). Besides their role in ATP synthesis, mitochondria regulate calcium homeostasis, thermogenesis, and apoptosis in response to nutrient fluctuations, hypoxia, cytokines, or changes in membrane potential; these processes are also tightly associated with ROS signaling regulation (Shadel and Horvath 2015). Excessive accumulation of mitochondrial ROS damages lipids, proteins, and nucleic acids, compromising mitochondrial function and releasing pro-apoptotic proteins that initiate cell death (Sena and Chandel 2012).

In addition to mitochondria, other endogenous sources of ROS include enzymes such as NADPH oxidases (NOX), myeloperoxidases (MPO), and xanthine oxidases, as well as organelles like peroxisomes and the ER (Orient et al. 2007; Aranda-Rivera et al. 2022b). Between them, the most involved in ROS overproduction in the kidney pathologies are the NOXs isoforms 2 and 4, which are overactivated in several models of renal damage (Hui et al. 2017; Bouchez and Devin 2019), likewise as their subunits (Gullans et al. 1988; Robben et al. 2009; Lu et al. 2018; Aparicio-Trejo et al. 2019). Interestingly, NOX4 is mainly localized in mitochondria (Aparicio‐Trejo et al. 2017), which favors pathological feedback of mitochondria and ROS production increase in renal damage (Gullans et al. 1988; Ortega-Domínguez et al. 2017; Aparicio-Trejo et al. 2022).

## ROS signaling and posttranslational modification

In recent years the role of ROS as a second signaling molecule has emerged in the cell signaling process, especially in the case of H_2_O_2_ and nitic oxide (NO) (Prem and Kurian 2021; Aranda-Rivera et al. 2022a). These ROS molecules can modify the activity, localization, stability, and association of several proteins by posttranslational modification of redox-sensitive amino acids, like cysteine (Cys) and methionine (Met) (Aparicio-Trejo et al. 2020a; Jedlicka et al. 2022). The Cys are particularly important in cell signaling due to their specificity and sensitivity for oxidation, which are highly dependent on the microenvironment and are influenced by neighboring charged amino acids (Rojas-Morales et al. 2019). In addition, the vast majority of H_2_O_2_-associated signaling has been related to reversible oxidation of specific thiol (–SH) of Cys (Fig. 1), giving as a result a wide range of oxidation states, ranging from sulfenic acid (R-SOH) to more oxidative states like sulfinic acid (R-SO_2_H) and sulfonic acid (R-SO_3_H) formation (Hall et al. 2009, 2013). Likewise, Cys can be oxidized to form disulfide bonds (S–S) or be conjugated with other ROS-associated molecules like NO forming S-nitrosothiol (R-SNO) or GHS forming S- glutathionylation (RS-SG), or H_2_S favoring persulfidation (R-SSH) (Tabei et al. 1983; Welch et al. 2001; Aparicio-Trejo et al. 2020b; Vringer and Tait 2023). The ROS-induced Cys modification can be classified into enzymatically reversible and non-enzymatically irreversible induced modifications; the reversible enzymatically-ones the most associated with redox signaling pathways in oxidative eustress conditions (Zhong et al. 2019; Aparicio-Trejo et al. 2020a, b). In contrast, the non-enzymatically irreversible ones mainly occur in higher ROS levels conditions, favoring the increase in classic oxidative markers and are usually not considered as part of redox signaling processes (Aparicio-Trejo et al. 2020a, b; Martínez‐Klimova et al. 2020). In addition, H_2_O_2_ can also react with Met, favoring methionine sulfoxide (MetO) formation, which is also reversed by the enzyme methionine sulfoxide reductase (MSR), being also considered as a redox signaling modification (Martínez-Klimova et al. 2019; Hogan et al. 2019). Finally, other amino acids like arginine (Arg), histidine (His), lysine (Lys), threonine (Thr), tyrosine (Tyr), and proline (Pro) are also modified by ROS (Tabei et al. 1983; Zhang et al. 2017).

The reversible Cys ROS modifications serve as a switch to control and modulate the enzymatic activity and protein function, activating or inactivating signaling pathways to respond to different stimuli and cell requirements (Aparicio-Trejo et al. 2020a, b). Because the redox states of these Cys thiols are regulated by the activity of thioredoxin (TRX) and GSH enzymatic systems and by the reductive power of NADPH (Fig. 1), the ROS signaling highly depends on NADPH/NADP^+^ and GSH/GSSG ratios (Tanada et al. 2017; Aparicio-Trejo et al. 2020a). As we previously discussed, the GSH/GSSG ratio changes depending on the organelle and cell compartment being usually the mitochondria matrix, the space with the higher ratio in the cell (Stallons et al. 2014; Hallan et al. 2017; Jung et al. 2024). Similarly, NADPH/NADP^+^ levels also change in the different cell compartments, which is linked with NADPH sources, being pentose phosphate pathway the principal contribution cytosol; meanwhile, in mitochondria, NADPH is generated by the nicotinamide nucleotide transhydrogenase (NNT) (Li et al. 2019b; Olona et al. 2022). This is particularly important in this organelle because NNT uses NADH to reduce NADP^+^ to NADPH (Corcoran and O’Neill 2016), linking energy metabolism and ROS regulation. Furthermore, the ROS signaling tightly coordinates bioenergetics, catabolism, and anabolism by NADH/NAD^+^ and NADPH/NADP^+^ systems that control the Cys switch oxidation in several proteins of the pathways. In fact, the changes in H_2_O_2_ levels, together with the NADH and NADPH, are the hub in the spatiotemporal response of redox signaling (Corcoran and O’Neill 2016; Tanada et al. 2017; Aparicio-Trejo et al. 2020a, b).

## Alterations of the Krebs cycle and OXPHOS in renal diseases

Several studies showed that the persistence of mitochondrial bioenergetics impairment is a key process in the genesis of AKI and CKD progression (Forbes and Thorburn 2018; Aparicio-Trejo et al. 2020a, b; Jiménez-Uribe et al. 2021; Lumpuy-Castillo et al. 2024). In this way, Krebs cycle activity is early affected after renal injury, leading to the accumulation and posterior excretion of many Krebs Cycle intermediates like citrate (Wei et al. 2014; Liu et al. 2017; Hallan et al. 2017), á-ketoglutarate (Tanada et al. 2017; Hallan et al. 2017; Harzandi et al. 2021), and succinate (Wei et al. 2014; Kamarauskaite et al. 2020; Jung et al. 2024), as a result in the reduction of the activity and expression of the correspondence enzymes (Wei et al. 2014; Liu et al. 2017, 2018; Hallan et al. 2017; Aparicio-Trejo et al. 2020a). In this way, renal biopsies of patients show lower Krebs cycle proteins mRNA levels and lower metabolites urinary concentrations (Stallons et al. 2014; Kang et al. 2015; Stadler et al. 2015; Hui et al. 2017; Hallan et al. 2017). Both oxidative stress and mitochondrial biogenesis reduction have been suggested as early inductors in the Krebs Cycle reduction (Hallan et al. 2017; Aparicio-Trejo et al. 2018; Liu et al. 2018; Bouchez and Devin 2019). As discussed in the next section, accumulating these metabolites triggers signaling pathways related to inflammatory regulation, especially in the case of succinate (Gullans et al. 1988; Robben et al. 2009; Lu et al. 2018). In addition, both ETC and OXPHOS capacity are strongly affected by renal damage. The renal activity of complex I (CI) usually decreases early on, after renal injury, and this reduction remains in an advanced stage of CKD (Aparicio‐Trejo et al. 2017; Ortega-Domínguez et al. 2017; Aparicio-Trejo et al. 2018, 2019, 2022). Similarly, complex II (CII), III (CIII), and IV (CIV) activities also decrease in several AKI and CKD models (Aparicio‐Trejo et al. 2017; Ortega-Domínguez et al. 2017; Aparicio-Trejo et al. 2018, 2019; Prem and Kurian 2021; Aranda-Rivera et al. 2022a; Jedlicka et al. 2022). Thus, the substantial reduction in ETC activity induces a drop in mitochondrial membrane potential (*ΔΨm*) (Aparicio‐Trejo et al. 2017; Rojas-Morales et al. 2019; Aparicio-Trejo et al. 2020a, b), especially in the proximal tubule (Hall et al. 2009, 2013). Therefore, as a result of the lower *ΔΨm*, the ATP synthase activity decreases (Aparicio‐Trejo et al. 2017; Ortega-Domínguez et al. 2017; Aparicio-Trejo et al. 2019, 2020a, b; Rojas-Morales et al. 2019), dropping mitochondrial ATP production (Aparicio‐Trejo et al. 2017; Aparicio-Trejo et al. 2019, 2020b; Jiménez-Uribe et al. 2021), which, together with the higher metabolic demand (Tabei et al. 1983; Aparicio-Trejo et al. 2020a) and decreasing O_2_ supply (Welch et al. 2001), leads to the renal ATP levels diminution in both AKI and CKD (Forbes and Thorburn 2018; Aparicio-Trejo et al. 2020a, b).

## Redox signaling and ß-oxidation impairment in renal diseases.

Proximal tubule cells are enriched in mitochondria, which is associated with the reabsorption of several molecules and electrolytes in this segment (Sekine and Endou 2013); the consumption of medium chain fatty acids (FA) is the principal substrate to provide acetyl-CoA and ATP production in this segment because glycolytic pathways are almost absent in the proximal tubule (Guder and Ross 1984; McDonough and Thomson 2012). Furthermore, gluconeogenic activity in the proximal tubule supplies energy substrate to distal segments of the nephron and is maintained by proximal ß-oxidation (ß-Ox) associated ATP production (Guder and Ross 1984; Zhao et al. 2017; Lumpuy-Castillo et al. 2024). Thus, mitochondrial ß-Ox provides the necessary ATP for all nephron segments’ energy demands.

The entry of long-chain FA to the cytoplasm can be regulated by different protein families: a cluster of differentiation-36 (CD36), FA-binding proteins (FABPs), and FA-transport proteins (FATPs), meanwhile medium and short-chain FA quickly enter through the membrane. Interestingly, several models of kidney diseases have been related to higher levels of lipid uptake proteins like CD36 and FABPs (Panduru et al. 2013; Souza et al. 2016; Nishi et al. 2019). Before ß-Ox, the FA is required to be activated by fatty acyl-CoA synthetase and internalized to the mitochondrion by carnitine palmitoyltransferase I (CPT1) and II (Samovski et al. 2023; Miner et al. 2023). Interestingly, CPT1 expression is also declared in CKD models, inducing lower mitochondrial ß-Ox activity (Yang et al. 2017; Aparicio-Trejo et al. 2020a, b; Gao and Chen 2022).

**Fig. 2:**
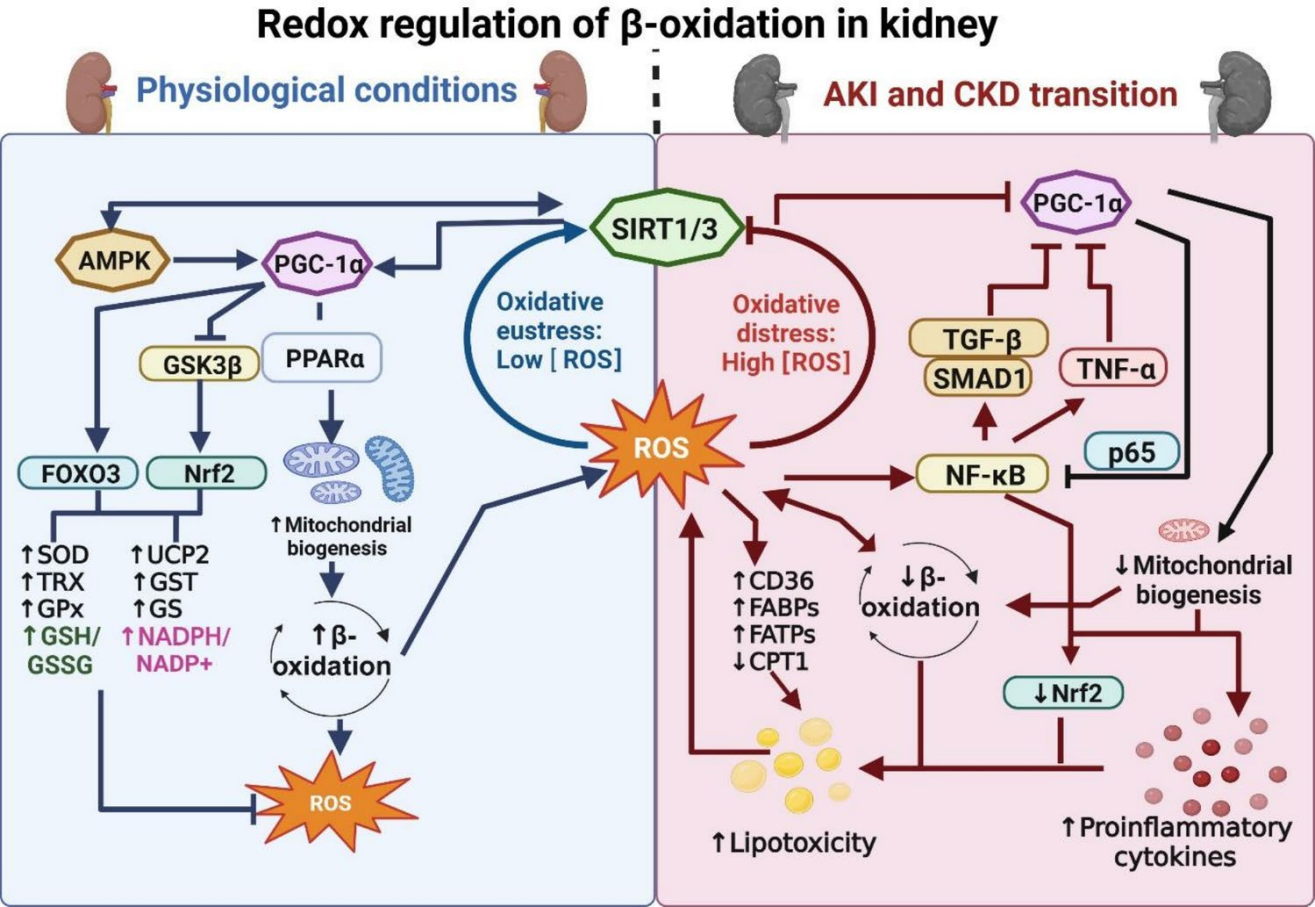
Redox regulation of mitochondrial β-oxidation in Kidney. Under physiologic conditions, ROS are produced in low concentration by mitochondrial processes like β-oxidation. These ROS favor the activation of SIRTs, which trigger the activation by deacetylation of the master kinase AMPK and the main mitochondrial biogenesis transcription factors. The increase in mitochondrial mass encourages an increase in mitochondrial ROS production. However, SIRT activation also promotes the transcription of antioxidant factors by the activation of FOXO3 and Nrf2, allowing the regulation of NADPH/NADP^+^ and GSH/GSSG ratios and the maintenance of the ROS levels in the cell redox tone corresponding to oxidative eustress. In contrast, in pathologic conditions, like AKI and CKD transition, high levels of ROS trigger the impairment in the SIRTs/AMPK/ PGC-1α axis. In addition, Nf-κB and TGF-β ROS activation further inhibit PGC-1α signaling. The reduction in mitochondrial mass and the increase in lipogenic and inflammatory factors favor the increase in renal lipotoxicity, promoting a higher increase in ROS levels and the establishment of the pathologic loop of damage. *AMPK* adenosine monophosphate-activated protein kinase, *CPT1* carnitine palmitoyltransferase 1, *CD3* cluster of differentiation 36, *GSH* reduced glutathione, *GSSG* oxidized glutathione, *GPx* glutathione peroxidase, *GST* glutathione S transferase, *GR* glutathione reductase, *GSK3β* glycogen synthase kinase-3 beta, *FABPB* fatty acid-binding protein, *FATP* fatty acid transport protein, *FOXO3* forkhead box O3A, *NADP*^*+*^ nicotinamide adenine dinucleotide phosphate oxidized form, *NADPH* nicotinamide adenine dinucleotide phosphate reduced form, *NF-κB* nuclear factor kappa-light-chain-enhancer of activated B cells, *Nrf2* nuclear factor erythroid 2, *PGC-1α* peroxisome proliferator-activated receptor γ coactivator 1α, PPARα peroxisome proliferators-activated receptor α, *SIRT* sirtuin, *SOD* superoxide dismutase, *TGF-β* transforming growth factor-beta, *TRX* thioredoxin, *UCP2* uncoupling protein 2. Figure created using BioRender.com

On the other hand, regulation of ß-Ox genes is regulated by peroxisome proliferators-activated receptor α (PPARα) (Yan et al. 2023) and peroxisome proliferator-activated receptor γ coactivator 1α (PGC-1α), the master regulator of mitochondrial biogenesis (Ye et al. 2024). The last one is highly modulated by adenosine monophosphate-activated protein kinase (AMPK)-sirtuins (SIRT) axis, enhancing mitochondria biogenesis (Jhuo et al. 2024). In this way, SIRTs and PGC-1α are between the principals’ redox hubs in the cells (Fig. 2), with a strong relation with the principal antioxidant factor, the nuclear factor erythroid 2 (Nrf2) (Singh et al. 2018; Sies et al. 2024). Therefore, the abundance and the activity of ß-Ox function enzymes and transcription factors are directly related to redox regulation (Yoboue et al. 2018; Sies et al. 2024).

In kidney diseases, alterations in ß-Ox can significantly impact kidney function and contribute to disease progression. This has been studied principally in CKD, where the downregulation of PGC-1α by transforming growth factor-beta (TGF-ß)/mothers against decapentaplegic homolog 1 (SMAD1) produces the diminish of PPARα abundance and, in consequence, the decrease of CPT1 (Gao and Chen 2022), which promotes apoptosis and fibrosis (Kang et al. 2015). In hypertensive renal injury, it was demonstrated that SIRT7 has a potential protective role in alleviating ferroptosis and lipid peroxidation through the Krüppel factor 15/ nuclear factor 2 (KLF15/Nrf2) signaling pathway (Li et al. 2022). Also, the upregulation of UCP2 increases the concentration in hypoxia-inducible factor 1-alpha (HIF-1α), which reduces PPARα and favors lipid depots. In AKI, SIRTs have an important role in ß-Ox regulation; meanwhile, the increase in SIRT5 impacts the concentration of PPARα, and the increase of SIRT3 stimulates it (Gao and Chen 2022). Besides, SIRT3 expression increases superoxide dismutase 2 (SOD) activity that restores redox balance decreasing oxidative stress and ferroptosis (Xie et al. 2024). Other authors suggest that the mitochondrial binding of PPARα with cyclophilin D inhibits its translocation to the nucleus, reducing ß-Ox in AKI (Jang et al. 2020). Lastly, in diabetic nephropathy, it has been mentioned that the advanced glycation end products decrease the activity of CPT1 (Gao and Chen 2022). These probably could be through the decrease of SIRT6, which has been associated with ferroptosis via Nrf2/glutathione peroxidase 4 (Gpx4) (Du et al. 2024) and is also necessary for ß-Ox enzymes expression (Gao et al. 2019). As is shown in Fig. 2, this data suggests a close relationship between SIRTs and ß-Ox regulation that involves a particular participation of PPARα, Nrf2, and antioxidant enzymes.

On the other hand, it is well known that ß-Ox dehydrogenases are one of the primary ROS sources in the mitochondria (Seifert et al. 2010). Furthermore, the redox regulation of ß-Ox enzymes, mainly by S-glutathionylation, mainly reduces their activity and OS production, allowing the regulation of redox homeostasis (Yoboue et al. 2018; Mailloux 2020). In addition, SIRTs participation in kidney disease impairment and redox imbalance has been previously widely studied (Xu et al. 2021; Ogura et al. 2021). However, the implication of SIRT mitochondrial ß-Ox in kidney diseases and its role in mitochondrial redox signaling has not been adequately elucidated.

On the other hand, it has been observed that the SIRT1/forkhead box O3A (FOXO3A) pathway is implicated in the activity regulation of PGC1a, PPARs, and pAMPK and its reduction promotes dysregulation in kidney lipid metabolism (Nguyen et al. 2017; Sies et al. 2024). Also, the decrease of SIRT1 increases ROS, driving from Gpx and SOD decrease through Nrf2/FOXO3A, working as an important therapeutic target by antioxidants like gastrodin or umbelliferone (Qiu et al. 2024). Besides, it has been mentioned that SIRT1 can increase the activity of CPT1A in vitro tubular renal cells through the STAT1/Twist1 pathway (Song et al. 2022), which is also activated by ROS-induced NF-κB signaling (Park et al. 2023).

In fructose-induced kidney injury, SIRT3 is downregulated; consequently, lipogenesis enzymes are over-expressed, and ß-Ox and antioxidant enzymes are decreased, which promotes lipid deposition and oxidative distress. However, the activation of SIRT3 by dioscin inhibits AKT/FOXO1, increasing CPT1 levels to diminish lipid deposition; it also enhances Nrf2/Keap1 signaling to increase SOD2 and glutathione s transferase (GST) levels, reducing oxidative distress (Qiao et al. 2018). Likewise, in mice with cisplatin-induced AKI, activating SIRT3 with honokiol, a polyphenol derived from the magnolia tree, improved ß-Ox, decreasing ROS levels and fibrosis; meanwhile, these protective effects were lost in SIRT3 knockout mice, emphasizing its participation (Li et al. 2020). These results suggested, together with our previous works (Aparicio-Trejo et al. 2020a, b; Ceja-Galicia et al. 2023) suggested, that the cellular protection pathways in renal diseases involved oxidative distress reduction by increasing antioxidant enzyme levels, as the increases ß-Ox enzymes, allowing the reduction in lipotoxic and restoring energetic balance. The ß-Ox increase protective mechanism is probably related to the NAD^+^/NADH rate, which is necessary for adequate mitochondrial function and SIRTs regulation. In quiescent cells, ß-Ox is used to regenerate NADH, which was observed through the inhibition of CPT1, which decreases ROS and induces cell dysfunction (Kalucka et al. 2018). Recently, the role of SIRT3 in insulin resistance and redox signaling was reviewed; they describe that the activation of SIRT3 maintains a proper NADH/NAD^+^ and GSH/GSSG ratio and improves CI function and ß-Ox (Cortés-Rojo et al. 2020), emphasizing the importance of NAD^+^ and glutathione redox status in ß-Ox activation and a possible application in kidney diseases. Likewise, our previous works in folic acid-induced AKI-to-CKD transition showed that mitochondrial S-glutathionylation and GSH/GSSG ratio reduction are strongly related to lower activity of ß-Ox and CI and SIRTs reduction in renal and cardiac tissues (Avila-Rojas et al. 2020; Li et al. 2022; Cuevas-López et al. 2023). On the other hand, the increase in H_2_O_2_ also participates in the fatty acid oxidation through the activation of mitochondrial redox-sensitive phospholipase A2 (iPLA2g/PNPLA8), which increases membrane FA availability for ß-Ox and, therefore, ATP generation (Jabůrek et al. 2024). This process induces insulin secretion in ß-cells and could promote the consumption of carbohydrates in other organs like the kidney. The increase in glycolysis may contribute to de novo lipogenesis and, therefore, the accumulation of lipids that may boost kidney disease (Milutinović et al. 2020). In fact, the increase of glycolytic pathways is a common pathologic pathway in AKI and CKD progression, tightly related to the deterioration of mitochondrial β-oxidation in the kidney (Lumpuy-Castillo et al. 2024). Besides, in kidney disease, CPT1A activity is decreased as well as ß-Ox and therefore elevates ROS production (Afshinnia et al. 2018; Aparicio-Trejo et al. 2020a; Lumpuy-Castillo et al. 2024). However, improved CPT1A activity or NAD^+^-mediated SIRT activation may contribute to reducing oxidative stress and fatty acid accumulation by β-oxidation stimulation.

In kidney diseases, several antioxidants have been proposed to improve renal functionality and decrease damage (Dehkordi et al. 2022). Most of them activate the Keap1/Nrf2 pathway; however, several of them also have protective effects by increasing PGC-1α and PPARα pathways, showing a dual effect by mitochondrial and antioxidant enzymes biogenesis promotion as well as activation (Negrette-Guzmán et al. 2015; Aparicio-Trejo et al. 2019; Avila-Rojas et al. 2020; Tanase et al. 2022; Ceja-Galicia et al. 2023). Thus, antioxidants could interplay with redox signaling and ß-Ox to reduce excess lipid and glucose damage by optimizing the OXPHOS system and, therefore, ROS reduction. Accordingly, in 5/6 nephrectomized rats, curcumin improves ß-Ox and mitochondria functionality in the kidney (Ceja-Galicia et al. 2022). Also, shen Shuai II, a Chinese herbal formula, which has antioxidant properties, has been used to treat CKD, and it was demonstrated its efficacy in increasing PPARα/NF-κB/NLRP3 pathway to reduce renal inflammation and promoting ß-Ox through CPT1 and 2 increase (Wang et al. 2024). In diabetic nephropathy induced with streptozotocin, resveratrol increases CPT1 and PPARα inhibiting mTOR (Li 2020) and AMPKα (Zhao 2020) autophagy pathways and reduces ROS increasing antioxidant enzymes (Salami et al. 2022). Besides, quercetin may improve fatty acid oxidation increasing PPARα in diabetic db/db mice kidneys (Guo et al. 2024), also increase GSH, and prevents ferroptosis (Wang et al. 2021). Catechins also prevent kidney damage by downregulating PPARγ/CD36 signaling (Patial et al. 2023), thus ß-Ox probably was stimulated. Thus, treating with antioxidants not only improves OXPHOS functionality and reduces ROS production but also may improve ß-Ox, making both systems more efficient to reduce ROS-induced cell damage and energetic crisis in kidney disease.

## Mitochondrial biogenesis

Mitochondrial biogenesis is the process by which preexisting mitochondria grow, and new mitochondrial biomolecules and the organelle are synthesized to meet the cell’s energy demands. This process enables adjustments in these organelles’ number, size, and shape according to cell type, metabolic needs, and physiologic or environmental conditions (Jornayvaz and Shulman 2010). The synthesis of new mitochondrial components relies on a precise interaction between nuclear-encoded and mitochondrial DNA (mtDNA) mitochondrial genes, regulated by several transcription factors and signaling pathways. This process is energetically expensive, requiring the assembly of numerous proteins and lipids in the corresponding membranes or organelle compartments. (Lee and Wei 2005). Mitochondrial biogenesis responds to internal and external stimuli, including hormones, second messengers like Ca^2+^, ROS signaling, metabolic balance, exercise, fasting, cold exposure, and inflammatory stress (Cherry and Piantadosi 2015).

Between the different factors, PGC-1α is considered the master regulator of mitochondrial biogenesis. This cofactor acts as a platform that recruits additional transcription factors, such as nuclear respiratory factors 1 (NRF1) and 2 (NRF2) (Kaur and Sharma 2022). NRF1 and 2 promote the synthesis of mitochondrial proteins encoded in the nucleus, which are subsequently imported into mitochondria to sustain their proliferation and ensure quality control. They also regulate the expression of key proteins involved in mtDNA replication and transcription, such as mitochondrial transcription factor A (TFAM), TFB1mt, TFB2mt, and Pol γ (Ploumi et al. 2017; Gureev et al. 2019). Subsequently, TFAM translocates to the mitochondrial matrix, where it stimulates mtDNA replication and mitochondrial gene expression, but also act as protection against mtDNA damage and oxidation (Kang et al. 2018).

Under low-energy conditions where the AMP/ATP ratio increases, thus PGC-1α can be activated by phosphorylation by AMPK, or the NAD^+^/NADH ratio description triggers PGC-1α deacetylation by SIRT1 activation. The PGC-1α activation favors its association with other factors like PPARγ, promoting their translocation to the nucleus and the over-expression of mitochondrial bioenergetics genes (Mineri et al. 2009; Popov 2020). In addition to PGC-1α, the PGC-1 family includes PGC-1β and PRC, which share sequence homology and regulate various metabolic pathways, such as cellular respiration, thermogenesis, and hepatic glucose metabolism (Ling et al. 2004; Scarpulla 2006; Maresca et al. 2013). While all these factors can stimulate mitochondrial biogenesis, each has a specific role. PGC-1α is primarily associated with gluconeogenesis, PGC-1β with fatty acid ß-Ox, and PRC with the coordination of nuclear and mitochondrial replication during the cell cycle (Ling et al. 2004; Maresca et al. 2013).

Other transcription factors also play a crucial role in mitochondrial biogenesis. For instance, estrogen-related nuclear receptors (ERRs), particularly ERRα, collaborate with PGC-1 to regulate genes associated with ß-Ox in aerobic tissues (Schreiber et al. 2004). In addition, CREB1, the initiator binding factor YY1, is involved in the basal regulation of respiratory genes and other genes related to energy metabolism (Spiegelman et al. 2000; Baar 2004; Madrazo and Kelly 2008). Among the critical genes for mitochondrial biogenesis, c-Myc, an activator of PGC-1, and MEF2A, a regulator of oxidative capacity in skeletal and cardiac muscles, stand out. The latter, activated by NRF-1, also stimulates genes involved in cell growth and survival (Morrish et al. 2003; Dang et al. 2005; Ramachandran et al. 2008).

Alterations in mitochondrial biogenesis may result in decreased mtDNA levels, triggering complex cellular responses that particularly affect genes related to the cell cycle and mitochondrial functions (Mineri et al. 2009). This highlights the critical importance of mitochondrial biogenesis in maintaining cellular homeostasis.

### Redox regulation of mitochondrial biogenesis in renal diseases

Mitochondrial biogenesis is crucial for maintaining renal function, as the kidneys require high energy production for tubular reabsorption (Bhargava and Schnellmann 2017). Impairments in mitochondrial biogenesis can lead to mitochondrial dysfunction, contributing to the development and progression of acute and chronic kidney diseases. Conversely, promoting mitochondrial biogenesis enhances metabolic pathways such as fatty acid oxidation and strengthens antioxidant defense mechanisms, which may mitigate kidney injury caused by aging, tissue hypoxia, and glucose or fatty acid overload (Weinberg 2011).

Mitochondrial biogenesis is typically activated by physiologic changes requiring increased ATP utilization rates. However, oxidative stress can also influence mitochondrial biogenesis (Lee et al. 2002; Nisoli et al. 2003; Piantadosi and Suliman 2012). The impact of ROS on mitochondrial biogenesis is concentration dependent. Low ROS levels activate signaling pathways that stimulate mitochondrial biogenesis, particularly in response to increased cellular energy demands (Gureev et al. 2019; Abu Shelbayeh et al. 2023). In this context, oxidative distress induces the expression of key factors such as NRF1, NRF2, PGC-1α, and PGC-1β, which are essential for regulating mitochondrial biogenesis and function (Baldelli et al. 2014; Gali Ramamoorthy et al. 2015; Gureev et al. 2019; Abu Shelbayeh et al. 2023). Furthermore, moderate ROS levels can promote survival mechanisms such as autophagy or mitophagy, eliminating damaged mitochondria and enabling cellular regeneration (Baldelli et al. 2014).

In contrast, elevated ROS levels can induce oxidative distress and damage to mitochondrial DNA and other cellular components, activating pro-apoptotic pathways that lead to programmed cell death. This process involves the release of cytochrome c into the cytosol and opening the mitochondrial permeability transition pore (Ala et al. 2022; Chen et al. 2022; Abu Shelbayeh et al. 2023).

**Fig. 3:**
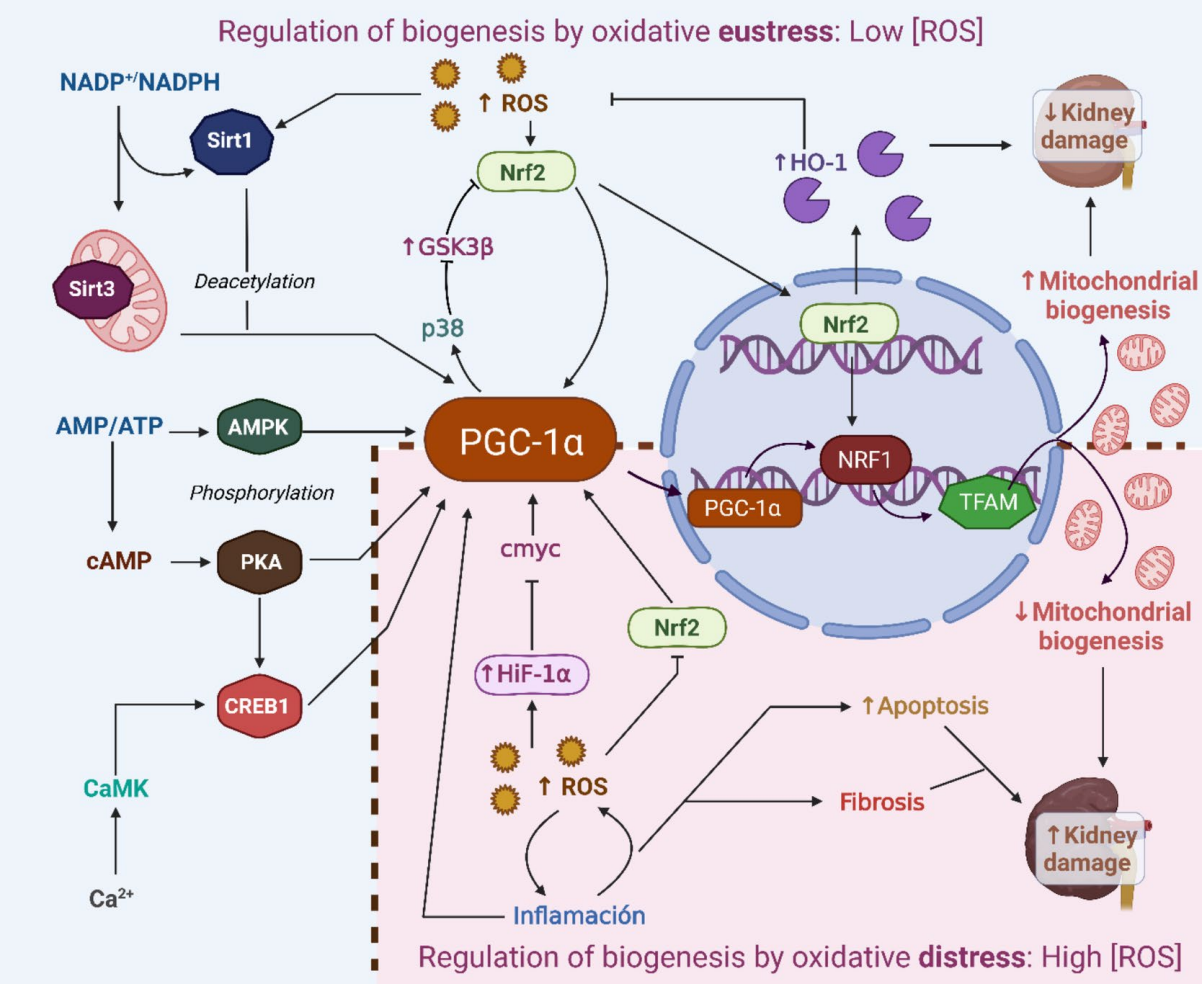
Redox regulation of mitochondrial biogenesis in renal injury. Mitochondrial biogenesis is commonly activated under low-energy conditions, characterized by an increased adenosine monophosphate/adenosine triphosphate (AMP/ATP) or oxidized/reduced nicotinamide adenine dinucleotide (NAD^+^/NADH) ratio. In this context, peroxisome proliferator-activated receptor gamma coactivator 1-alpha (PGC-1α) is activated through phosphorylation by AMP-activated protein kinase (AMPK) or deacetylation via sirtuin 1 (SIRT1). PGC-1α subsequently activates nuclear respiratory factor 1 (NRF1), which stimulates mitochondrial transcription factor A (TFAM) and other factors responsible for mitochondrial DNA (mtDNA) replication. Reactive oxygen species (ROS) can also influence mitochondrial biogenesis. A moderate level of ROS can activate the nuclear factor erythroid 2-related factor 2 (Nrf2), which positively regulates NRF1. In addition, PGC-1α can upregulate Nrf2 through the p38/glycogen synthase kinase beta 3 (GSK3β)/Nrf2 pathway. This moderate ROS production is associated with increased mitochondrial biogenesis, likely as a compensatory response that protects against renal damage. In contrast, excessive ROS levels reduce Nrf2 activity while increasing hypoxia-inducible factor 1-alpha (HIF-1α), leading to a decrease in mitochondrial biogenesis. These conditions exacerbate inflammation, further inhibiting biogenesis and creating a vicious cycle that promotes ROS overproduction. This scenario is associated with a higher risk of apoptosis and fibrosis, ultimately aggravating renal damage. *Ca*^*2*^*⁺* calcium ion, *CaMK* calcium/calmodulin-dependent protein kinase, *cAMP* cyclic adenosine monophosphate, *Cmyc* cellular myelocytomatosis oncogene, *CREB1* cAMP response element-binding protein 1, *HO-1* heme oxygenase-1, *PKA* protein kinase A. Figure created using BioRender.com

In kidney diseases, the dual effect of ROS on mitochondrial biogenesis has been observed (Fig. 3). A study in mice demonstrated that selenium deficiency results in renal damage characterized by increased ROS levels. However, a compensatory response was observed through elevated SIRT1 and PGC-1α expression, indicating an attempt to counteract mitochondrial dysfunction (Lai et al. 2021). Nevertheless, in the context of renal diseases, ROS elevation is more commonly associated with oxidative distress and detrimental effects on mitochondrial function and renal health rather than stimulation of mitochondrial biogenesis (Fernández Agudelo and Zeledón Corrales 2020).

In renal diseases such as diabetic nephropathy, ischemia–reperfusion injury, and CKD, oxidative distress is linked to the downregulation of mitochondrial biogenesis via factors such as NRF1, PGC-1α, and TFAM (Wang et al. 2020; Liao et al. 2022; Chen et al. 2022; Prem et al. 2023; Gong et al. 2023). This disruption affects mitochondrial function and promotes pro-inflammatory and fibrotic processes, exacerbating renal dysfunction (Liao et al. 2022).

During inflammatory processes, oxidative distress further inhibits mitochondrial biogenesis. As discussed in the next section, higher ROS have been shown to promote the production of pro-inflammatory cytokines and chemokines that suppress this process. For example, TNF-α, a key pro-inflammatory cytokine, acts as a catabolic agent that interferes with mitochondrial respiration, inhibiting biogenesis and directly impacting regulatory molecules such as PGC-1α (Remels et al. 2010). In cardiomyocytes, TNF-α stimulates the binding of the NF-κB p65 subunit to PGC-1α, potentially disrupting downstream gene activation mediated by both molecules (Boucher 2010). Notably, in AKI and CKD, increased inflammation is widely associated with elevated ROS levels and reduced mitochondrial biogenesis, as evidenced by decreased markers such as PGC-1α (Chen et al. 2022; Salari et al. 2022; Siddhi et al. 2022; Elkhoely 2023; Shahmohammadi et al. 2023; Gong et al. 2023; Fan et al. 2024). Moreover, mitochondrial mass reduction appears to correlate with NF-κB activation and overproduction of TNF-α and nitric oxide, exacerbating cellular damage. This effect has been observed in various experimental models of renal injury, including lipopolysaccharide (LPS)-induced damage, AKI, and CKD (Wang et al. 2020; Li et al. 2021; Salari et al. 2022; Lotfi et al. 2023). Thus, the interplay between oxidative stress, inflammation, and mitochondrial biogenesis represents a central axis in the pathophysiology of renal injury, underscoring the need for therapeutic strategies targeting these processes.

In addition, ROS modulates the stability and activity of HIF-1α, which plays a critical role in cellular responses to hypoxia and is closely associated with oxidative stress. The impact of ROS on HIF-1α influences key processes such as cellular inflammation, metabolism, and stress responses (Piantadosi and Suliman 2012). While HIF-1α activation under oxidative stress conditions may act as an initial protective mechanism, chronic activation can lead to metabolic and signaling alterations that perpetuate cellular damage (Li et al. 2019a). In specific contexts, HIF-1α stabilization can suppress mitochondrial biogenesis by inhibiting the transcriptional activity of c-Myc (Zhang et al. 2007). In cisplatin-induced AKI, increased HIF-1α levels are accompanied by oxidative stress and reduced PGC-1α expression (Gong et al. 2023). This study also revealed that HIF-1α activation increased heme oxygenase-1 (HO-1) levels, which were associated with ferroptosis, a type of iron-dependent cell death. This relationship highlights the complexity of HIF-1α and oxidative stress roles in renal injury, particularly in their interaction with mitochondrial biogenesis and metabolic adaptations.

A promising approach to managing kidney diseases involves modulating oxidative stress. Recent research has shown that controlling ROS production through antioxidants can restore mitochondrial biogenesis and improve renal function, providing an innovative therapeutic strategy for these pathologies (Guo et al. 2015). For instance, in a 5/6 nephrectomy CKD model, curcumin restored PGC-1α expression, promoting mitochondrial regeneration and reducing renal damage (Wang et al. 2020). Similarly, sulforaphane treatment in malic acid-induced renal damage improved mitochondrial biogenesis and reduced oxidative stress, mitigating renal insufficiency (Briones-Herrera et al. 2020).

Activation of antioxidant pathways, such as Nrf2 signaling, has also demonstrated beneficial effects in experimental models of kidney diseases. For example, scutellarin modulates the Nrf2/PPAR-γ/PGC-1α pathway, reducing ROS, promoting mitochondrial biogenesis, and alleviating inflammation (Shahmohammadi et al. 2023). This protective effect improves mitochondrial function and prevents the progression of LPS-induced AKI. Similarly, lycopene treatment enhances PGC-1α expression and activates Nrf2 in LPS-induced nephrotoxicity (Salari et al. 2022).

In diabetic nephropathy, 4-octyl itaconate (4-OI), an itaconate derivative, improves renal function and reduces tubular damage in db/db mice. This occurs through Nrf2 activation and PGC-1α-mediated mitochondrial biogenesis promotion (Shao et al. 2024).

The Nrf2/ARE signaling pathway regulates mitochondrial biogenesis (Gureev et al. 2019). It has been demonstrated that PGC-1α activates Nrf2 through the inhibition of GSK3β, establishing a PGC-1α/p38/GSK3β/Nrf2 signaling pathway that connects both transcriptional regulators of mitochondrial function (Puigserver et al. 2001; Choi et al. 2017). Nrf2 and PGC-1α may also form a positive feedback loop, where Nrf2 directly increases PGC-1α expression. Conversely, Nrf2 suppression reduces mitochondrial biogenesis and PGC-1α expression in various tissues (Athale et al. 2012; Baldelli et al. 2013; Whitman et al. 2013; Joe et al. 2015). Therefore, the interaction between Nrf2 and PGC-1α is critical for mitochondrial biogenesis regulation, and its dysfunction could contribute to the development and progression of kidney diseases.

Other compounds have also demonstrated renoprotective effects by enhancing mitochondrial biogenesis and reducing oxidative stress, highlighting their therapeutic potential in renal diseases. For instance, melatonin exerts a protective effect in diabetic nephropathy by activating the AMPK/SIRT1 pathway, which stimulates autophagy, improves mitochondrial biogenesis, and reduces apoptosis (Siddhi et al. 2022). Similarly, liraglutide protects against gentamicin-induced AKI by activating the PGC1α/PKA/CREB and Notch1/Hes-1 pathways, thereby reducing oxidative stress, inflammation, and apoptosis (Elkhoely 2023).

On the other hand, astragalin (AG), an antioxidant flavonoid, has shown renoprotective effects in diabetes by inhibiting aldose reductase (ALR2)-induced oxidative stress. Its action, mediated by the AMPK/PGC-1α pathway, optimizes mitochondrial quality control by balancing mitochondrial fission and fusion processes and promoting mitochondrial biogenesis, thus preventing diabetic kidney damage (Sun et al. 2023). In addition, eprosartan has proven effective in an ischemia/reperfusion-induced kidney injury model by enhancing mitochondrial function by activating the Sirtuin 1/PGC1α/Sirtuin 3 pathway. This mechanism reduces ROS generation, preserves intracellular ATP levels, and decreases inflammation and apoptosis, offering a promising therapeutic strategy against ischemia/reperfusion stress (Lotfi et al. 2023).

In conclusion, ROS signaling and mitochondrial biogenesis are closely linked to kidney diseases. In contrast, moderate ROS and oxidative eustress support cellular repair; elevated ROS concentrations and distress exacerbate mitochondrial damage and induce inflammation and apoptosis. On the other hand, compounds such as curcumin, sulforaphane, melatonin, liraglutide, astragalin, and eprosartan have demonstrated renoprotective effects by modulating ROS- mitochondrial biogenesis interactions, highlighting their therapeutic potential. Table 1 summarizes the studied models of kidney injury, the effects of oxidative stress, and mitochondrial biogenesis alterations, providing a comprehensive overview of their relationship and potential interventions.

**Table 1:** Summary of renal damage models, oxidative stress effects, mitochondrial biogenesis alterations, and treatments used

Model	Oxidative Stress	Biogenesis	Other	Treatment	References
Acute Kidney Injury: Cecal ligation and puncture	↓ CAT ↓ SOD ↓Nrf2	↓ TFAM ↓ Mfn2 ↓PGC-1α ↑ Drp-1 ↓ ΔΨm ↓ ATP	↑ BUN ↑Serum creatinine ↑ TNF-α, ↑ IL-1β ↑ HMGB1	Hydrogen at 67%	(Pei et al. 2024)
Renal tubular injury: Diabetic nephropathy	↓Nrf2 ↓ SOD1 ↓ SOD2 ↑ ROS	↓PGC-1α ↓TFAM ↓mtDNA	↑ BUN ↑ Albumin ↑ NGAL ↑ Apoptosis	4-octyl itaconate	(Shao et al. 2024)
Cardiorenal syndrome: subtotal nephrectomy (5/6) to induce chronic kidney disease	↑ ROS ↑ NOX-2 ↑ NOX-4	↓ AMPK ↑ p-PI3K ↑ p-mTOR ↓ TFAM ↓ p-SIRT1 ↓ PGC-1α	↑ BUN ↑Serum creatinine ↑ Proteinuria ↑ Renal artery reactive index ↑ Inflammation ↑ Angiotensin II ↑ AT1R	Empagliflozin	(Yang et al. 2024)
Acute Kidney Injury: Lipopolysaccharide	↑ MDA ↓ GPx ↓ SOD ↓ CAT ↓ Nrf2 ↓ HO-1 ↑ ROS	↓ ΔΨm ↓ mtDNA ↓ PGC-1α	↑ BUN ↑Serum creatinine ↑ Inflammation ↑ TNFα ↑ IL-1β ↑ IL-6 ↑ iNOS ↓ IL-10	Malvidin	(Fan et al. 2024)
Hypertensive kidney disease: 5/6 nephrectomy	↑ NOX-1 ↑ NOX-2 ↑ p-ERK1/2 ↑ p-p38 ↑ p- JNK	↑ Autophagy ↑ Mitophagy ↓ PGC-1α ↓ mit-CytoC ↓ Mfn2	↑ BUN ↑Serum creatinine ↑ Proteinuria ↑ Apoptosis (Bax, caspase 3) ↓ Bcl-2	Dapagliflozin-Entresto	(Ko et al. 2023)
Acute Kidney Injury: Cisplatin	↓ GSH/GSSG ↓ SOD ↑ MDA ↑ Fe^2+^ ↑ HO-1	↓ UCP2 ↓ Mfn2 ↓ PGC-1α	↑ BUN ↑Serum creatinine ↑ CX3CL1 ↑ Ferroptosis ↑ Inflammation ↑ Macrophage infiltration ↑ HIF1A	–	(Gong et al. 2023)
Ischemia–reperfusion injury + high-fat diet	↑ TBARS ↓ GSH/GSSG ↓ CAT ↓ SOD ↓ GPx	↓ PGC-1α ↓ TFAM ↓ Polg ↑ Dnm1 ↑ Fis 1 ↓ Mff ↓ Mfn1 ↓ Mnf2 ↑ Mitophagy ↑ Drp1	↑ BUN ↑ Serum creatinine ↑ KIM-1 ↓ Na–K ATPase	–	(Prem et al. 2023)
Diabetic kidney disease	↑ MDA ↑ ALR2 ↑ NADP^+^/ NADPH ↓ GSH/GSSG ↓ CAT ↓ SOD ↑ ROS	↓ Mitochondrial mass ↓ ΔΨm ↓ PGC-1α ↓ TFAM ↓ NRF1 ↓ ERRα ↓ ATP5F1 ↓ NDUFV1 ↓ Cox7a ↓ CPT1 ↑ ACC2 ↓ ACADM ↓ PPAR α ↓ Drp-1 ↓ Fis 1 ↓ OPA1 ↓Mfn1 ↓Mfn2	↑ Fasting glucose ↑ Random glucose ↑ TG ↑ TCHO ↑ LDL-C ↑ Serum creatinine ↑ Apoptosis	Astragalin	(Sun et al. 2023)
Ischemia/reperfusion renal injury	↑ MDA ↑ NO ↓ GSH/GSSG ↓ CAT ↓ SOD ↓ GPx	↓ PGC-1α ↓ SIRT 1 ↓ SIRT 3	↑ Serum Creatinine ↑ Urea ↑ NF-κB ↑ COX2 ↑ Apoptosis ↑ Inflammation ↑ klotho	Eprosartan	(Lotfi et al. 2023)
Gentamicin-induced kidney injury	↑ MDA ↓ GSH/GSSG ↓ SOD ↓ GPx	↓ PGC-1α ↓ PKA/CREB	↑ Serum Creatinine ↑ Urea ↑ Notch1/Hes-1 ↑ Inflammation (IL-1β, TNF-α) ↑ Apoptosis (Bax, Caspase-3)	Liraglutide	(Elkhoely 2023)
Kidney injury: Lipopolysaccharide	↑ ROS ↑ MDA ↓ SOD ↓ Nrf2 ↓ HO-1	↓ Mitochondrial function ↓ ΔΨm ↓ PGC-1α ↓ PPARγ ↑ MPO	↑ Inflammation (TLR4, NF-κB, TNF-α) ↓ IL-10 ↑ Serum Creatinine ↑ BUN ↑ Serum Cystatin C	Scutellarin	(Shahmohammadi et al. 2023)
Diabetic nephropathy	↑ ROS ↑ MDA ↓ SOD ↓ GSH	↓ Mitochondrial function ↓ PGC-1α ↓ TFAM ↓ Autophagy	↑ Inflammation (TNF-α, IL6) ↑ Apoptosis (Caspase-3, Caspase-9)	Melatonin	(Siddhi et al. 2022)
Sepsis-associated acute kidney injury	↑ ROS ↑ MDA ↓ SOD	↓ ATP ↓ ΔΨm ↓ PGC-1α ↓ TFAM ↓ mtDNA	↑ Apoptosis ↑ Inflammation	–	(Chen et al. 2022)
LPS-induced nephrotoxicity	↑ ROS ↑ MDA ↓ SOD ↓ Nrf2	↓ Mitochondrial Function ↓ ΔΨm ↓ PGC-1α ↑ MPO	↑ Serum Creatinine ↑ BUN ↑ Serum cystatin C ↑ Inflammation (NF-κB, TLR4)	Lycopene	(Salari et al. 2022)
Unilateral ureteral obstruction	↑ ROS ↓ SOD ↑ 4-HNE ↓ TRX2 ↑ NOX4 ↓ SIRT3	↓ Mitochondrial function ↓ PGC-1α ↓ TFAM ↓ NRF1 ↓ SIRT1	↑ Serum Creatinine ↑ BUN ↑ Renal Fibrosis ↑ Uric Acid	Fluorofenidone	(Liao et al. 2022)
Ischemia–reperfusion injury	↑ ROS ↓ SOD ↑ 4-HNE ↓ TRX2 ↑ NOX4 ↓ SIRT3	↓ Mitochondrial function ↓ PGC-1α ↓ TFAM ↓NRF1 ↓ SIRT1	↑ Serum Creatinine ↑ BUN ↑ Uric Acid ↑ Renal Fibrosis	Fluorofenidone	(Liao et al. 2022)
Ischemia–reperfusion injury	↑ ROS ↑ MDA ↓ Nrf2	↓ ΔΨm ↓ ATP ↓ PGC-1α ↓ TFAM ↓ Autophagy	↑ Renal Damage ↑ BUN ↑ Serum Creatinine ↑ Inflammation (TNF-α, IL1β) ↑ Apoptosis	Empagliflozin	(Ala et al. 2022)
Acute Kidney Injury: Ischemia/reperfusion	↓ HO-1 ↑ 8-OHdG	↓ TFAM ↓ ATP5α	↑ Inflammation (NF-κB , TNF-α, IL1β, IL6) ↑ BUN ↑ Serum Creatinine ↑ NGAL	–	(Li et al. 2021)
Diabetic nephropathy	↑ MnSOD2	↑ Drp1 ↑ PGC-1α	↑ Jagged1 ↑ Notch1 ↑ pro-caspase-3	–	(Jing et al. 2021)
Kidney damage: selenium deficiency	↑ MDA	↓ COXIV ↓ Cytochrome c ↑ SIRT1 ↑ PGC-1α	↑ Serum creatinine ↑ Urinary protein/creatinine ratio ↑ Renal inflammation ↑ Swollen mitochondria	–	(Lai et al. 2021)
Diabetic nephropathy	↓GSH ↓ CAT ↓SOD ↑TBARS	↓PGC-1α ↓NRF1 ↓mtDNA/nDNA	↑ Glucose ↑ ALP	Syringic acid	(Rashedinia et al. 2021)
Chronic kidney disease: 5/6 Nephrectomy	↑ MDA ↓ SOD ↓ CAT ↓ GPx	↓ PGC-1α ↓ TFAM ↓ NRF1 ↓ mtATP ↓ COXIV	↑ Serum creatinine ↑ Proteinuria ↑ Inflammation (NF-κB, IL-1β, IL-6) ↑ GSK-3β	Curcumin	(Wang et al. 2020)
Fanconi Syndrome: Maleic Acid	**↑**ROS ↑ 4-HNE	↓ PGC-1α ↓ ΔΨm) **↓** Complex I activity ↑ Mitochondrial fragmentation ↑ Mitophagy	↑ Proteinuria ↑ Plasma GPx ↑ NGAL ↑ KIM-1	Sulforaphane	(Briones-Herrera et al. 2020)
Chronic kidney disease: Subtotal nephrectomy	↓ Citrate synthase ↑ H_2_O_2_	↓ PGC-1α ↓ ATP synthase ↓ COX IV ↓ Mitochondrial mass	Not evaluated	AST-120	(Nishikawa et al. 2015)

*4-HNE* 4-hydroxynonenal, *8-OHdG* 8-Hydroxy-2’-deoxyguanosine, *CADM* Acyl-CoA dehydrogenase medium chain, *ACC2* acetyl-CoA carboxylase 2, *ADNn* ADN nuclear, *ALP* alkaline phosphatase, *ALR2*: aldose reductase, *AT1R* angiotensin II type 1 receptor, *ATP* adenosine triphosphate, *ATP5F1* ATP synthase F1 subunit beta, *ATP5α* ATP synthase subunit alpha, *Bax* Bcl-2-associated X protein, *Bcl-2* B-cell lymphoma 2, BUN blood urea nitrogen, *CAT* catalase, *COX IV* cytochrome c oxidase subunit IV, *COX2* cyclooxygenase-2, *Cox7a* cytochrome c oxidase subunit 7A, *CPT1* carnitine palmitoyltransferase 1, *CREB* cAMP response element-binding protein, *CX3CL1* Fractalkine, *Dnm1* Dynamin-1, *Drp-1* dynamin-related protein 1, *ERK1/2* extracellular signal-regulated kinases 1 and 2, *ERRα* estrogen-related receptor alpha, *Fe*^*2+*^ ferrous ion, *Fis1* mitochondrial fission 1 protein, *GPx* glutathione peroxidase, *GSH* glutathione, *GSK-3β* glycogen synthase kinase 3 beta, GSSG glutathione disulfide, *H*_*2*_*O*_*2*_ hydrogen peroxide, *HIF1A* hypoxia-inducible factor 1-alpha, *HMGB1* high-mobility group box 1, *HO-1* heme oxygenase-1, *IL-10* interleukin-10, *IL-1β* Interleukin-1 beta, *IL-6* interleukin-6, *iNOS* inducible nitric oxide synthase, *JNK* c-Jun N-terminal kinase, *KIM-1* kidney injury molecule-1, *LDL-C* low-density lipoprotein cholesterol, *MDA* malondialdehyde, *Mff* Mitochondrial fission factor, *Mfn1-2* mitofusins 1 and 2, *mit-CytoC* mitochondrial cytochrome c, *MnSOD2* manganese superoxide dismutase 2, *MPO* myeloperoxidase, *mtDNA* mitochondrial DNA, *mTOR* mechanistic target of rapamycin, NADP^+^ nicotinamide adenine dinucleotide phosphate, *NADPH* nicotinamide adenine dinucleotide phosphate (reduced form), *NDUFV*1 NADH dehydrogenase [ubiquinone] flavoprotein 1, *NF-κB* nuclear factor kappa-light-chain-enhancer of activated B cells, *NGAL* neutrophil gelatinase-associated lipocalin, *NO* nitric oxide, *NOX *NADPH oxidase, *NRF1* nuclear respiratory factor 1, *Nrf2* nuclear factor erythroid 2–related factor 2, *OPA1* optic atrophy 1, *p38* p38 mitogen-activated protein kinases, *PGC-1α* peroxisome proliferator-activated receptor gamma coactivator 1-alpha, *PI3K* phosphoinositide 3-kinases, *PKA* protein kinase A, *Polg* DNA polymerase gamma, *PPARα* Peroxisome proliferator-activated receptor alpha, *PPARγ* peroxisome proliferator-activated receptor gamma, *ROS* reactive oxygen species, *SIRT3* sirtuin 3, *SIRT1* Sirtuin 1, *SOD* superoxide dismutase, *TBARS* thiobarbituric acid reactive substances, *TCHO* total cholesterol, *TFAM* mitochondrial transcription factor A, *TG* triglycerides, *TLR4* toll-like receptor 4, *TNF-α* Tumor necrosis factor-alpha, *TRX2* thioredoxin 2, *UCP2* uncoupling protein 2, *ΔΨm* Mitochondrial membrane potential

## NLRP3 and mitochondria ROS in renal diseases

**Fig. 4:**
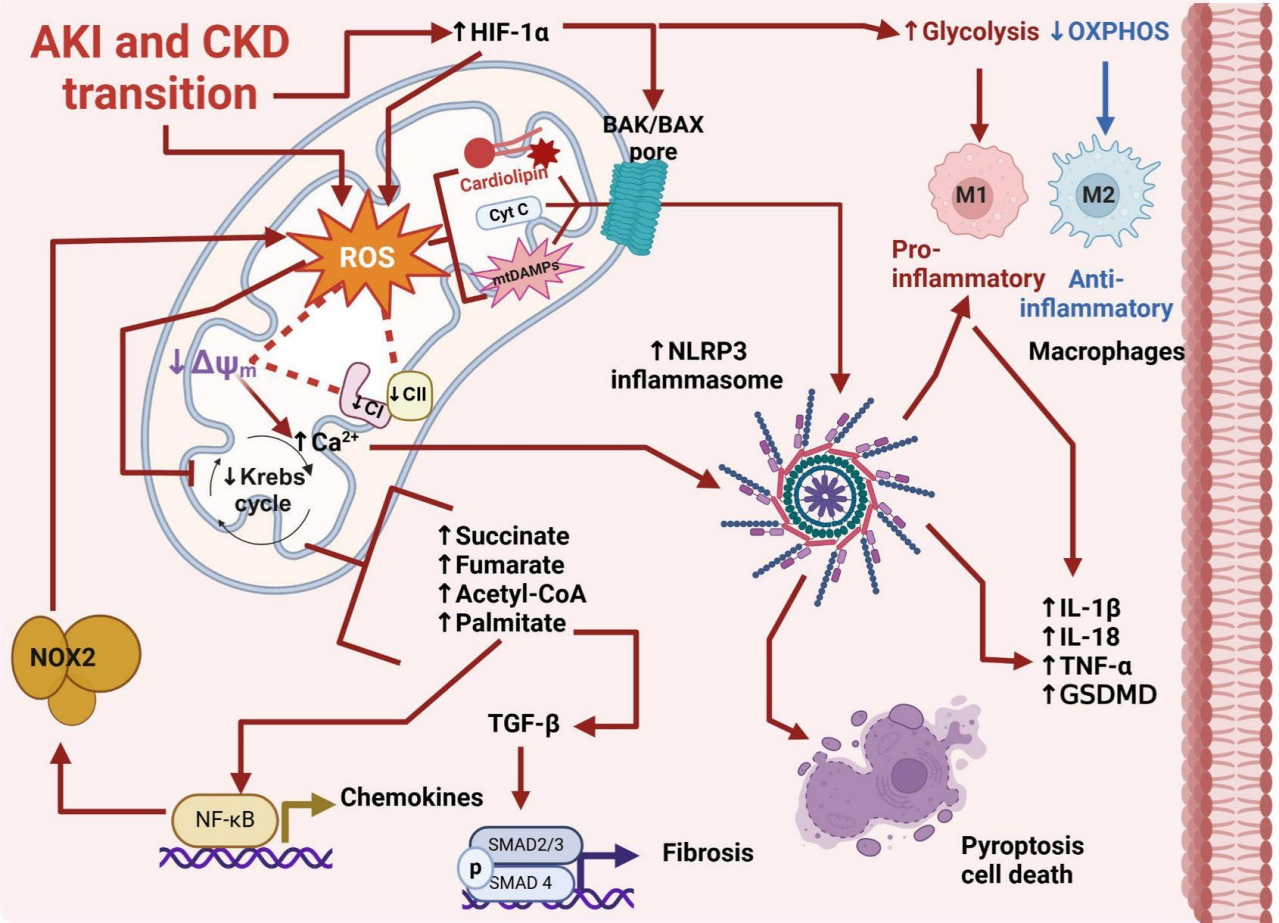
NRLP3 ROS-induced signaling in renal disease. AKI and CKD transition are characterized by mitochondrial ROS overproduction, strongly linked to CI/II impairment and mitochondrial membrane potential (*ΔΨm*) depolarization. These three factors promote the mitochondrial damage and the formation on BAK/BAX pore, allowing the cytosolic releases of Cyt c, mtDAMPs, oxidized cardiolipin and other factors that promote NLRP3 assembled an activation. Furthermore, the mitochondrial impairment and ROS production increase impairment mitochondrial pathways like Krebs cycle, favoring the increase of intermediate metabolites like palmitic acid and succinate, strongly associated with the promotion of pro inflammatory and fibrotic pathways. Five more mitochondrial ROS-induced impairment promote Ca^2+^ homeostasis disruption, enhancing NOX and NRLP3 activation, favoring chemokines production and in last the cell death by processes such as pyroptosis. *BAK* Bcl-2 homologous antagonist/killer, BAX BCL-2-associated X protein, *CI* complex I, *CII* complex II, *Cyt C* cytochrome C, *GSDMD* gasdermin D, *HIF-1α* hypoxia-inducible factor 1-alpha, *IL-1β* interleukin 1 beta, *IL-18* interleukin 18, *mtDAMP* mitochondrial damage-associated molecular patrons, *NOX2* NADPH oxidase2, *NRLP3* NLR family pyrin domain containing 3, *NF-κB* nuclear factor kappa-light-chain-enhancer of activated B cells, *Nrf2* nuclear factor erythroid 2, *PGC-1α* peroxisome proliferator-activated receptor γ coactivator 1α, *PPARα* peroxisome proliferators-activated receptor α, *TNF-α* tumor necrosis factor-alpha, *TGF-β* transforming growth factor-beta. Figure created using BioRender.com

Mitochondria is a central hub in bioenergetics and redox regulation. However, this organelle is also a point of convergence for several inflammatory and cell death-inducing signaling (Zhong et al. 2019; Vringer and Tait 2023), which is strongly regulated by metabolic and redox fluxes (Martínez-Klimova et al. 2019; Martínez‐Klimova et al. 2020; Aparicio-Trejo et al. 2022). The impairment in the Krebs Cycle may induce inflammatory pathways through the promotion of mitochondrial ROS production (Fig. 4); thus, renal succinate and fumarate increase in unilateral renal obstruction and hypoxia-induced CKD models lead to an increase in ROS production (Liu et al. 2017; Zhang et al. 2017; Li et al. 2019b; Hogan et al. 2019). Interestingly, the reduction of mitochondrial metabolism and increased aerobic glycolysis are characteristic of M1 proinflammatory macrophage activation in an NRLP3-associated pathway (Olona et al. 2022).

In this way, ischemia-induced HIF-1α activation triggers succinate accumulation favoring macrophage activation, mediated by the NLRP3 pathway (Fig. 4), resulting in the rise of IL-1β and IL-18 secretion (Corcoran and O’Neill 2016; Olona et al. 2022). Furthermore, succinate dehydrogenase (CII) inhibition by itaconic acid can reduce the NLRP3 activation and the release of pro-inflammatory molecules (Corcoran and O’Neill 2016), suggesting a robust metabolic regulation of this inflammatory pathway. In this way, renal succinate accumulation in the diabetic kidney has been shown to reduce mitochondrial fatty acid β-oxidation (Wang et al. 2022), inducing an increase in the acetyl-CoA/CoA ratio. This excess acetyl-CoA can be excreted as acetylcarnitine; remarkably, CKD patients show significantly increased serum acetylcarnitine concentrations, which correlates with reduced renal function (Wang et al. 2022).

In CKD, acetyl-CoA increase is related to lipid intermediates and lipid derivatives accumulation in the kidney (Szeto et al. 2016; Nishi et al. 2019). From early CKD stages, nephron segments like proximal tubules increase their lipid levels (Kang et al. 2015; Nishi et al. 2019) and the CD36 levels (Souza et al. 2016; Nishi et al. 2019), a transporter also associated with inflammatory pathways induction (Okamura et al. 2009; Aparicio-Trejo et al. 2020a; Martínez‐Klimova et al. 2020). CKD lipotoxicity increases fatty acid levels in the plasma and kidneys, especially palmitic acid (Szeto et al. 2016; Ly et al. 2017; Ceja-Galicia et al. 2022). Palmitic acid has been shown to inhibit AMPK signaling, reducing mitochondrial function and increasing mitochondrial ROS production to favor the NLRP3 inflammasome activation and caspase-1, IL-1b, and IL-18 production (Wen et al. 2011; Ly et al. 2017). This agrees with recent works that suggest that early mitochondrial ETS impairment triggers the renal reduction in β-oxidation (Aparicio-Trejo et al. 2020a, b; Jiménez-Uribe et al. 2021).

Advanced stages of CKD are also characterized by the downregulation of β-oxidation enzymes in the kidneys (Wang et al. 2005; Herman-Edelstein et al. 2014; Szeto et al. 2016; Feng et al. 2017), which induce inflammation in proximal tubules and glomeruli (Yang et al. 2017; Aparicio-Trejo et al. 2020a). Interestingly, we previously showed in folic acid and 5/6 nephrectomy-induced CKD models that the reduction in palmitic acid β-oxidation is related to mitochondrial H_2_O_2_ production, coupling reduction, and fission induction, increasing inflammatory markers in the kidney (Aparicio-Trejo et al. 2020a, b). The higher fatty acid synthesis in CKD may also induce NLRP3 pathway activation by the fatty acid synthase (FASN) induction, an enzyme that is upregulated in pro-inflammatory macrophages type I, leading to upregulation of the expression of the inflammasome components: NLRP3, caspase 1 and pro-IL-1β (Olona et al. 2022). Interestingly, both mRNA silencing messenger and chemical inhibition of FASN suppressed NLRP3 activation and inhibited the macrophage expression of pro-inflammatory cytokines like IL-1β (Moon et al. 2015). Likewise, the reduction of ETS activity has been linked to increased mitochondrial ROS production and oxidative distress (Correa et al. 2013; Aparicio‐Trejo et al. 2017; Hui et al. 2017; Aparicio-Trejo et al. 2019). In this way, CI and CIII reduction in CKD (Aparicio-Trejo et al. 2020a, b) has been related to the NLRP3-NFkB pathway activation (Gong et al. 2016; Kim et al. 2018; Swanson et al. 2019; Aparicio-Trejo et al. 2020a; Nam et al. 2022). The imiquimod inhibition of complex I and quinone oxidoreductase administration activates the NLRP3 pathway by the increase in ROS levels (Groß et al. 2016).

Bioenergetics alterations and lipotoxicity in kidneys also induce Ca^2+^ dysregulation in CKD (Ly et al. 2017; Martínez‐Klimova et al. 2020), favoring NRLP3 activation (Swanson et al. 2019). The increase in fatty acid levels like palmitate induces the release of Ca^2+^ from the ER, resulting in mitochondrial Ca^2+^ overload (Ly et al. 2017), mediated by the mitochondrial Ca^2+^ uniporter (MCU) complex. Under stress conditions and due to lower ΔΨm, mitochondrial Ca^2+^ extrusion systems are insufficient to reduce Ca^2+^ concentration (Giorgi et al. 2018a, b). These produce mitochondrial Ca^2+^ accumulation and mitochondrial membrane transition pore (MPTP) opening, allowing the release of mitochondrial factors to the cytosol (Sandhir et al. 2017; Giorgi et al. 2018b). Mitochondrial depolarization also induces the recruitment of BAX and BAK and pore formation in the outer mitochondrial membrane (MOM) (Flores-Romero et al. 2023). However, under caspase inhibition, the release of BAX-BAK pore induces NRLP3 inflammasome activation and increases NF-κB, leading to proinflammatory cytokine production (White et al. 2014; Giampazolias et al. 2017; Vringer and Tait 2023). In kidney injury, the palmitate-induced Ca^2+^ levels dysregulation leads to podocyte damage, which can be prevented by mitoTEMPO administration, highlighting the mitochondrial ROS role in Ca^2+^-mediated MPTP (Ly et al. 2017). Although NRLP3 activation attributes the MTPT opening to *ΔΨ* loss by enhanced mitochondrial ROS production (Swanson et al. 2019), it is essential to remember that in renal pathologies, the reduction in mitochondrial CI and CII activity by itself induced ΔΨ depolarization (García-Arroyo et al. 2017; Aparicio‐Trejo et al. 2017; Ortega-Domínguez et al. 2017; Briones-Herrera et al. 2018; Aparicio-Trejo et al. 2019; Rojas-Morales et al. 2019), making this organelle more susceptible to MTPT opening and BAX and BAK recruitment.

In CKD, damaged mitochondria are a convergence point for inflammatory pathways (Kang et al. 2015; Kim et al. 2018; Martínez‐Klimova et al. 2020). In the NRLP3 pathway, mitochondria act as docking sites for inflammasome assembly by the mitochondria-associated membranes (MAMs) (Missiroli et al. 2018). Oxidized cardiolipin is associated with MAMs where it binds to NLRP3 and caspase 1; these interactions are necessary for inflammasome activation (Iyer et al. 2013; Swanson et al. 2019), interestingly renal damage increases cardiolipin oxidation (Szeto et al. 2016; Dang et al. 2017). Likewise, MAVS and mitofusin 2 (a mitochondrial fusion protein) form a complex that allows NLRP3 localization in mitochondria and its activation (Yuk et al. 2020), suggesting a link between mitochondrial dynamic and inflammasome activation. This is particularly important in CKD, where bioenergetic alterations trigger mitochondrial shift to fission and impairment in mitophagy flux, allowing the accumulation of damaged mitochondria (Aparicio-Trejo et al. 2018, 2020b, 2022; Martínez‐Klimova et al. 2020). In fact, defective mitophagy in macrophages strongly exacerbated NLRP3 activation, showing that mitochondria removed by this mechanism is a key regulator of inflammasome activity (Swanson et al. 2019; Yuk et al. 2020). Likewise, mitophagy also removes mitochondrial DAMPs like mtDNA and oxidized cardiolipin, which trigger TLR9 and promoteenal and heart failure (Zhong et al. 2019; Nie et al. 2020).

In addition, to mtDNA, mitochondrial permeabilization in the kidney releases several immunogenic factors like cardiolipin, N-formyl peptides, cytochrome c, SMAC, succinate, and ROS, among others (Xiang et al. 2020; Vringer and Tait 2023). Later, NLRP3 inflammasome activation increases the production of chemokines but also leads to pyroptosis, an inflammatory type of regulated cell death. This mitochondrial permeabilization by BAX and BAK triggers caspase 1 and 3 activations, which cleave gasdermin family proteins, especially D (GSDMD). Then, GSDMD binds to phosphatidylserine in the cell membrane inner and oligomerizes, forming a pore in the plasma membrane (Vringer and Tait 2023; Flores-Romero et al. 2023). This mechanism allows the release of mitochondrial DAMPs, cytokines, and chemokines to the extracellular medium in a non-conventional secretory way (Wu et al. 2021; Olona et al. 2022; Vringer and Tait 2023; Flores-Romero et al. 2023). Likewise, in CKD, Ca^2+^ dysregulation and lipid levels increase induce the secretion of extracellular vesicles (EVs) by renal cells (Dini et al. 2020; Aparicio-Trejo et al. 2022). These EVs can transport mitochondrial components, DAMPS, chemokines, and cytokines to induce inflammation and metabolic reprogramming in distant recipient cells (Aparicio-Trejo et al. 2022). Thus, both pyroptosis and EVs pathways allow the import of mitochondrial DAMPs and chemokines to peripheral tissues, spreading inflammation at a systemic level.

## ER and mitochondria in kidney disease

The ER is related to metabolic and biosynthetic cellular processes and intraorganellar communication (Marchi et al. 2014). The dysfunctional ER is associated with the onset and advancement of kidney illnesses, evidenced by elevated levels of ER stress markers in AKI and CKD (Ohse et al. 2006; Mostafa et al. 2020; Shu et al. 2022). Also, persistent ER stress can induce inflammation and fibrosis; as we previously discussed, both are closely related to kidney disease progression (Shu et al. 2022; Wu et al. 2023). In addition, mitochondria and ER communication is important for Ca^2+^ cellular homeostasis; for that reason, a synergistic relationship and the disruption or malfunction of the organelle communication have an important role in kidney disease progression (Rizzuto et al. 2009; Wu et al. 2023). Communication between mitochondria and ER occurs through the mitochondria-associated ER membrane (MAM, also known as mitochondria ER contact sites). These membranes regulate lipid transfers, and Ca^2+^ homeostasis and play an important role in mitochondria dynamics, autophagy, inflammation, and fibrosis process (Marchi et al. 2014; Wu et al. 2023). As referred to previously, MAMs integrity is associated with renal disease progression.

### ER stress and unfolded protein response (UPR)

**Fig. 5:**
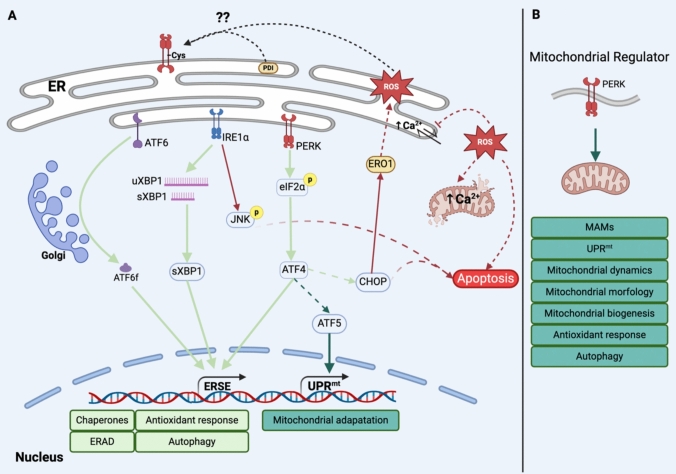
Unfolded Protein Response (UPR), PERK as a mitochondrial regulator. **A** An adaptative response is activated during ER stress, through three sensors (IRE1, ATF6, and PERK) to reestablish ER homeostasis. However, a prolongate response activates other pathways that converge in apoptosis via mitochondrial also, this induces oxidative distress which has implications on the ER membrane and oxidation of Cys residues of protein related to protein synthesis (chaperons, PDI), calcium homeostasis (SERCA, IP3R), and UPR sensors like PERK. (B) PERK through the PERK/ATF4/CHOP pathway signaling diverse pathways related to mitochondrial function and mitochondrial homeostasis was recently appointed “mitochondrial regulator”. *ATF6* activating transcription factor 6, *ATF6f* activating transcription factor 6 fragment, *IRE1α* inositol-requiring enzyme 1 α, *PERK* protein kinase R-like ER kinase, *uXBP1* X-box binding protein 1 unspliced, *sXBP1* X-box binding protein 1 spliced, *JNK *JUN N-terminal kinase, *eIF2α* eucaryotic translation initiation factor 2α, *ATF*4 activating transcription factor 4, *ATF5* activating transcription factor 5, *CHOP* C/EBP homologous protein, *ERO1* endoplasmic oxidoreductase 1, *PDI* protein disulfide isomerase, *ERSE* ER stress response element, *ERAD* ER-associated degradation, *UPR*^*mt*^ mitochondrial unfolded protein response, *MAMs* mitochondria-associated ER membrane. Figure created using https://BioRender.com

The ER is a single membrane organelle, a highly dynamic and fluid membrane system, representing the largest organelle (Maly and Papa 2014). The function of ER includes protein and lipid synthesis, Ca^2+^ regulation, and organelle crosstalk (Wu et al. 2023). Diverse types of stress may disrupt ER homeostasis, a process referred to as ER stress, characterized by the accumulation of unfolded or misfolded proteins in the lumen of the ER, as well as an imbalance of Ca^2+^ homeostasis and lipid synthesis (Casas-Martinez et al. 2024). The ER has a protective mechanism to reestablish ER homeostasis; the most studied include the unfolded protein response (UPR) (Malhi and Kaufman 2011). Mammals have three UPR sensors; the first is the inositol-requiring enzyme 1 (IRE1), IRE1α is the main isoform, which presents phosphorylase activity and endoribonuclease activity, causes mRNA degradation and the alternative splicing of X-box binding protein 1 (XBP1s) upregulated the expression of the machinery for protein synthesis and factors related to protein degradation (Maly and Papa 2014). The second sensor is the activating transcription factor 6 (ATF6), a transcription factor activated in the Golgi, upregulates XBP1 and chaperones enhancing the ER folding capacity and reestablishing ER homeostasis (Malhi and Kaufman 2011). Finally, the third sensor is the protein kinase R-like ER kinase (PERK) a kinase protein that phosphorylates eucaryotic translation initiation factor 2α (eIF2α), inhibiting protein translation and inducing the translation of specific genes affecting several cellular pathways, including upregulated chaperon synthesis, antioxidants pathways (Nrf2, GST, HO-1, etc.), autophagy and mitochondrial biogenesis (Casas-Martinez et al. 2024). Also, all three sensors induce pathways related to the apoptosis process depending on the duration and severity of the ER stress (Fig. 5) (Casas-Martinez et al. 2024).

High levels of ER stress must induce the activation of inflammatory and apoptosis pathways. The overactivation of ribonuclease activity of IRE1 reduces levels of specific microRNA, that normally repress pro-apoptotic and pro-inflammatory proteins (Maly and Papa 2014). This facilitates an elevated thioredoxin-interacting protein (TXNIP) level, activating the NLRP3 inflammasome and Caspase-1. Also, IRE1, through phosphorylation of apoptosis signal-regulating kinase 1 (ASK1) allows the activation of c-Jun N-terminal kinase (JNK), resulting in the activation of BIM and inhibition of Bcl-2 converging on the mitochondrial apoptotic pathway (Malhi and Kaufman 2011). In fact, IRE1/JNK signaling was associated with the transition of AKI to CKD (Liang et al. 2022).

The PERK pathway was related to ER stress contributing to kidney disease progression in cisplatin-induced CKD (Shu et al. 2022). Also, PERK contributes to the development of renal fibrosis induced by trimethylamine N-oxide, a gut metabolite implicated in CKD, associated with the Akt/mTOR pathway and NLRP3 and Cas-1 activation (Shu et al. 2021; Kapetanaki et al. 2021). On the other hand, the inhibition of PERK suppresses fibrosis in a model of tunicamycin-induced CKD, a classical ER stress inducer (Shu et al. 2021).

### Redox regulation of UPR members

The ER has an oxidative environment favoring the folding proteins, through the formation of disulfide bridges, which contributes to the redox state generating H_2_O_2_ as a constitutive ROS production (Casas-Martinez et al. 2024). The ER-oxidizing environment is greater than the cytosol, so a low GSH/GSSG ratio in ER is important for disulfide reduction and maintenance of ER homeostasis. An excess of GSH in the ER can induce UPR by compromising oxidative protein folding (Eletto et al. 2014a). Also, in response to endogenous and exogenous stressors, the ER can increase its protein folding capacity, inducing redox environment changes that can cause ER dysfunctions (Eletto et al. 2014a).

The protein disulfide isomerase (PDI), a lumen ER protein, introduces disulfide bonds to nascent proteins and mediates oxidative folding, reducing PDI (Malhi and Kaufman 2011). The reoxidation of PDI is principally through the endoplasmic oxidoreductase 1 (ERO1α), an enzyme that produces H_2_O_2_ as a side-product of catalysis. Moreover, the nitrosative stress causes the inactivation of PDI through nitrosylation, inactivating its active-site cysteine residue and promoting the ER Ca^2+^ efflux (Nakato et al. 2015).

The activation of UPR in response to oxidative stress is mediated by members of the PDI family, PDI family A member 5 (PDIA5), and PDIA6 interact with the sensors of UPR (Eletto et al. 2014a). PDIA5 is suggested to cleave the disulfide bonds present in ATF6, ATF6 form monomers and oligomers on Cys467 and Cys618 residues in the ER lumen domine, allowing the ATF6 processing to an active transcription factor (Higa et al. 2014). Adversely, PDIA6 negatively regulates IRE1 and PERK. IRE1 has Cys residues indispensable for signaling (Cys148 and Cys332), PDIA6 can direct binding in the luminal domine of IRE1 via a disulfide bond, also ROS can affect these Cys residues affecting the oligomerization of IRE1 consequently its activation (Eletto et al. 2014b; Kranz et al. 2017). Sulfenylation of Cys residues of active kinase loop of IRE1 inhibiting UPR induction and promoting p38 activation inducing the antioxidant response pathway (Nrf2) (Hourihan et al. 2016). On the other hand, PDI family A member 3 (PDIA3) regulates positively PERK signaling (Kranz et al. 2017).

In a rat model of diabetic nephropathy, the PDIA3 protein level increase and the Piroxiredoxin-1 protein level decreased was associated with disturbance in the homeostasis of the ER stress, inflammation, fibrosis, and dysregulation on antioxidant capacity (Hsu et al. 2015). High secretory level of PDIA3, associated with ER stress, was related to the establishment and progression of renal fibrosis (Dihazi et al. 2013). The absence of PDIA6 was recently demonstrated to enhance ER stress and ferroptosis in proximal tubule epithelial cells (Kim et al. 2024). Nevertheless, the examination of Cys residues in PERK and IRE1 has yet to be assessed in the context of renal illness (Koike et al. 2025).

### PERK response to ER and mitochondrial stress

PERK is close to MAM contacts; also, those contacts contain proteins involved in Ca^2+^ homeostasis, lipid transfer, redox balance, and mitochondrial homeostasis (Casas-Martinez et al. 2024). PERK phosphorylates eIF2α at serine 51 promoting ATF4 transcription; his regulates genes related to protein synthesis, redox homeostasis, amino acid metabolism, autophagy, and apoptosis (Malhi and Kaufman 2011). ATF4 is a feedback loop of eIF2α, through the activation of C/EBP homologous protein (CHOP) transcription factor, which induces the transcription of growth arrest and DNA damage 34 (GADD34) protein which forms a holophosphatase complex to dephosphorylate eIF2α (Harding et al. 2009).

Also, the integrated stress response (ISR) is characterized by the phosphorylation of eIF2α, through three kinases apart of PERK, induced by oxidative stress, heat stress, alcohol, virus, or nutrient deprivation (Humeau et al. 2020). The ISR is induced in response to mitochondrial stress, and the eIF2α-ATF4-CHOP pathway promotes the expression of ATF5 to resolve proteotoxic and oxidative stress. ATF5 is preferentially induced by an increase in mitochondrial dysfunction; this is a part of the UPR mitochondrial, reducing proteotoxic and oxidative stress, reestablishing mitochondrial homeostasis, and inducing fission (Drp1) (Almeida et al. 2022; Casas-Martinez et al. 2024). Although the PERK pathway is the principal UPR sensor related to mitochondrial homeostasis, under some conditions of renal disease, the transcription factor ATF6 decreases the expression of PPARα and downregulates the expression of genes related to mitochondrial beta-oxidation, leading to fibrosis (Casas-Martinez et al. 2024).

The importance of PERK in mitochondria lies in its localization at the MAMs. During the ER stress, PERK can be reversible oxidate at Cys216, allowing the interaction with ERO1α narrowing MAM formation and increasing Ca^2+^ flux into the mitochondria regulating bioenergetics (Bassot et al. 2023). Also, CHOP can activate the transcription of ERO1α promoting an oxidative ER environment (Marciniak et al. 2004). On the other hand, the adaptative response of UPR modulates mitochondrial biogenesis through the PERK-Nrf2 pathway (Casas-Martinez et al. 2024). As a part of the UPR mitochondrial, the PERK-ATF4-CHOP pathway promotes mitophagy and biogenesis (PGC1α, TFAM, and NRF1). Also, CHOP is a regulator of mitochondrial dynamics because it upregulates the expression of Fis1 and OPA as a protective effect (Casas-Martinez et al. 2024).

For all those reasons, PERK was described as a regulator of mitochondrial capacity because it can modulate calcium flux, MEMs formation and function, mitochondrial dynamics, mitochondrial cristae formation through UPR mitochondrial, and UPR as antioxidant response and autophagy (Fig. 5) (Perea et al. 2023; Casas-Martinez et al. 2024).

### Redox regulation of ER and mitochondria in calcium homeostasis

Impairment in redox homeostasis can be detrimental to renal disease, so maintenance of ER and mitochondrial homeostasis and MAMs is important to avoid disease progression (Casas-Martinez et al. 2024). The activation level of UPR regulates Ca^2+^ transport to mitochondria via MAM interactions. Since an adaptative UPR response can increase Ca^2+^ import, metabolism, and dynamics of mitochondrial, a prolonged or maladaptive UPR response causes excessive calcium release and import to mitochondria, inducing apoptosis (Wu et al. 2023).

The ER is the main intracellular Ca^2+^ store, the Sarcoendoplasmic reticulum Ca^2+^ ATPase (SERCA) influx Ca^2+^ into the ER-dependent on ATP, whereas the Ca^2+^ leak from the ER is controlled by the inositol 1,4,5-triphosphate receptor (IP3R) and Ca^2+^ leak pores. The ER chaperones like calreticulin, GRP78, GRP94, and enzymes as PDI help to maintain the Ca^2+^ levels to the physiological range (Rizzuto et al. 2009; Kaufman and Malhotra 2014).

The ER stress results in loss of Ca^2+^, induced by lower SERCA activity, altered IP3R, or increased passive Ca^2+^ leak. SERCA and IP3R are subject to redox regulation; SERCA is inhibited by sulfoxidation at Cys674 and is activated by glutathionylation at Cys674 by NO (Eletto et al. 2014a). Redox regulation of IP3R on Cys residues changes Ca^2+^ release activity, its oxidation by PDIA15 and the reduction by ERdj5 caused the activation and the inactivation of IP3R activity, respectively (Fujii et al. 2023). In addition, ROS overproduction causes Ca^2+^ depletion through IP3R, affecting chaperon function and folding protein; ROS can oxidate membrane lipids that affect ER membrane fluidity and avoid conformational changes necessary for SERCA activity and other ER membrane proteins (Fujii et al. 2023).

CHOP expression and phosphorylation of eIF2α and JNK modulate Ca^2+^ flux between the mitochondria and the ER, modulating mitochondrial metabolism and inducing the opening of mPTP and activation of apoptotic pathways. Mainly, the PERK-ATF4-CHOP arms regulate the Ca^2+^ flux because the induction of ERO1 by CHOP induces the activation of IP3R, favoring the leak of Ca^2+^ (Eletto et al. 2014a). The MAM has an important function in Ca^2+^ flux between the mitochondria and ER, and proteins related to Ca^2+^ flux are present in MAM regions as a PERK.

PDI is affected by perturbation in Ca^2+^ homeostasis because diminished Ca^2+^ levels prevent its oxidoreductase activity from perturbing the redox environment in the ER (Fujii et al. 2023).

All of these are important because components of the TCA cycle require Ca^2+^ binding to their optimal function; these enzymes are pyruvate-, a-ketoglutarate-, and isocitrate- dehydrogenase (Rizzuto et al. 2009). The production of ATP is necessary for the function of several ATP-dependent ER proteins, such as SERCA, to maintain the Ca^2+^ concentrations as Ca^2+^-dependent chaperons necessary for protein folding (Kaufman and Malhotra 2014).

## Final remarks

The high dependence of renal function on mitochondrial homeostasis, together with the central role of this organelle in cellular signaling, makes mitochondrial impairment a key in renal disease development. This organelle, ER, and peroxisomes mainly form a redox triangle in charge or regulation of cell homeostasis. Under physiologic conditions, the ROS produced in this organelle, especially H_2_O_2_ acts as a second messenger that, together with the change in the NADPH/NADP^+^ and GSH/GSSG ratios, induce post-translational modification of the protein modifying their activity, localization, and/or stability. These allow the correct maintenance of processes like mitochondrial biogenesis by the SIRTs/AMPK/ PGC-1α axis, allowing the maintenance of mitochondrial β-oxidation, Krebs cycle, and calcium regulation. ROS and calcium also act in the correct communication between mitochondria and ER, which is key to maintaining the correct homeostasis in renal cells, especially in the proximal tubule. Interestingly, several proteins involved in mitochondrial and ER processes are sensitive to Cys modification induced by ROS, making these pathways highly dependent on redox tone.

As has been widely described, AKI and CKD progression are characterized by an excess of ROS concentration that overwhelms physiologic levels. This does not inhibit the physiologic redox pathways by unspecific oxidations of the protein another biomacromolecule, like the involved in biogenesis and energetic mitochondrial metabolism. But additionally allowing the activation of other redox pathways, traditionally related to pathologic processes, like inflammation, fibrosis or ER stress. These pathways are activated by proteins like NF-κB, NRLP3, HIF1-α, and TGF-β contributing to reduced mitochondrial metabolism, inducing pro-inflammatory cytokines and cell death factors elevation and inducing oxidative and ER stress, characteristic of renal diseases.

Although much evidence has been shown that the redox regulation of these pathways is presented in AKI and CKD, information about the specific redox modification in the protein of these pathways is still scarce, specifically in renal cells. Therefore, it is necessary to do a deeper study of the redox-regulated proteins, especially in renal mitochondria and ER, to better understand the ROS in the renal homeostasis maintenance and in the development of processes involved in renal diseases. This may allow the development of new or better treatments to advance one of the pathologies with the greatest increase and prevalence worldwide today, renal diseases.

## Data Availability

Not applicable.
